# Identification of cuproptosis-related patterns and construction of a scoring system for predicting prognosis, tumor microenvironment-infiltration characteristics, and immunotherapy efficacy in breast cancer

**DOI:** 10.3389/fonc.2022.966511

**Published:** 2022-09-23

**Authors:** Wei Li, Xingda Zhang, Yanbo Chen, Da Pang

**Affiliations:** ^1^ Harbin Medical University Cancer Hospital, Harbin, China; ^2^ Heilongjiang Academy of Medical Sciences, Harbin, China

**Keywords:** Breast cancer, cuproptosis, drug sensitivity, tumor microenvironment, immunotherapy

## Abstract

**Background:**

Cuproptosis, a recently discovered refreshing form of cell death, is distinct from other known mechanisms. As copper participates in cell death, the induction of cancer cell death with copper ionophores may emerge as a new avenue for cancer treatment. However, the role of cuproptosis in tumor microenvironment (TME) cell infiltration remains unknown.

**Methods:**

We systematically evaluated the cuproptosis patterns in The Cancer Genome Atlas (TCGA) database in breast cancer (BRCA) samples based on 10 cuproptosis-related genes (CRGs), and correlated these patterns with the prognosis and characteristics of TME cell infiltration. A principal component analysis algorithm was used to construct a cuproptosis score to quantify the cuproptosis pattern in individual tumors. Further, the relationships between the cuproptosis score and transcription background, clinical features, characteristics of TME cell infiltration, drug response, and efficacy of immunotherapy were assessed.

**Results:**

Two distinct cuproptosis patterns with distinct prognoses were identified; their TME characteristics were found to be consistent with the immune-excluded and immune-inflamed phenotypes, respectively. The cuproptosis patterns in individual patients were evaluated using the cuproptosis score based on the cuproptosis phenotype-related genes, contributing to distinguishing biological processes, clinical outcome, immune cell infiltration, genetic variation, and drug response. Univariate and multivariate Cox regression analyses verified this score as an independent prognostic predictor in BRCA. A high cuproptosis score, characterized by immune activation, suggests an inflamed tumor and immune-inflamed phenotype with poor survival and a low cuproptosis score, characterized by immune suppression, indicates a non-inflamed tumor and immune-excluded phenotype with better survival. Significant differences were observed in the IC50 between the high and low cuproptosis score groups receiving chemotherapy and targeted therapy drugs. In the two immunotherapy cohorts, patients with a higher cuproptosis score experienced considerable therapeutic advantages and clinical benefits.

**Conclusions:**

This study is the first to elucidate the prominent role of cuproptosis in the clinical outcome and the formation of TME diversity and complexity in BRCA. Estimating cuproptosis patterns in tumors could help predict the prognosis and characteristics of TME cell infiltration and guide more effective chemotherapeutic and immunotherapeutic strategies.

## Introduction

Female breast cancer (BRCA) has the highest global cancer incidence and is the leading cause of cancer related deaths among women. According to the latest global cancer statistics, an estimated 2.3 million new female BRCA cases were diagnosed, while approximately 685,000 died from it in 2020 (1). Standardized treatment approaches for BRCA include surgery, chemotherapy, hormonal therapy, radiotherapy, and targeted therapy. Although considerable progress has been made in these approaches and the associated mortality reduced, a significant proportion of patients still experience recurrence or metastasis, including those who had received comprehensive treatment in the early stages (2). Clinically, the prognosis and treatment strategy for BRCA are traditionally determined according to the clinical tumor-node-metastasis (TNM) stage and molecular subtype, which are limited by its high heterogeneity and thus may not achieve good outcomes (3–5). Therefore, unveiling the genomic characteristics underlying BRCA is vital for the development of clinically applicable models for predicting prognosis and assessing the therapeutic response, which could further improve precise and individualized treatment.

Cuproptosis is a newly discovered form of cell death that depends on mitochondrial respiration and is distinct from known cell death mechanisms such as apoptosis and pyroptosis (6). Copper accumulates in the cells and directly binds to lipoylated components of the tricarboxylic acid cycle. This leads to abnormal aggregation of the lipoylated protein and the subsequent loss of the iron-sulfur cluster protein, which together result in proteotoxic stress and ultimately cell death. Moreover, some copper ionophores have been demonstrated to increase intracellular copper levels to induce tumor cell death (7).

Immunotherapy, especially immune checkpoint blockade (ICB), has revolutionized the treatment of cancer, particularly advanced-stage cancers. Nevertheless, although a subset of patients experiences dramatic and long-term disease regression, the majority of patients do not benefit from these treatments (8). The tumor microenvironment (TME), mainly composed of tumor, immune, and stromal cells; fibroblasts; endothelial cells; pericytes; extracellular matrix elements; and diffusible cytokines and chemokines secreted from cancer and stromal cells, has been demonstrated to play crucial roles in tumor development and progression, immune escape, and the response to both traditional treatment and immunotherapy (9). BRCA has strong immunogenicity; it is infiltrated by a variety of immune cells, which affects its development (10–13). In BRCA, the complexity and heterogeneity of the TME affect the response to both chemotherapy and immunotherapy (14). Predicting ICB responses based on TME cell-infiltrating characteristics is crucial in improving the effectiveness of existing ICBs and developing new immunotherapy strategies (15, 16). Hence, a deeper analysis of the heterogeneity and complexity of TME landscapes has the potential to provide more advanced prognostic biomarkers, identify different tumor immune phenotypes, and improve the ability to guide and predict immunotherapy responses.

Recently, the most investigated regulated cell death mechanisms include apoptosis, necroptosis, pyroptosis, ferroptosis, PANoptosis, and autophagy, all of which play crucial roles in modulating the TME and determining the clinical outcomes of cancer therapeutic approaches. Combining their inducers and ICBs results in synergistically enhanced antitumor effects (17, 18). The copper ionophore disulfiram (DSF) has exhibited anticancer activity in BRCA (19). The DSF-copper complex has demonstrated a powerful ability to inhibit cancer cell growth and reverse drug resistance. Furthermore, the combination of DSF and copper has a greater anticancer effect than monotherapy alone. The DSF-copper complex inhibited BRCA cell growth by targeting the reactive oxygen species level, ubiquitin proteasome system, and NPL4 (19–21). Compared to traditional treatment modalities, this complex demonstrated better selectivity. For example, it inhibited the proteasomal activity and selectively induced apoptosis in malignant MDA-MB-231 and MCF10DCIS.com BRCA cells, but not in normal, immortalized MCF10A breast cells (20). More importantly, the DSF-copper complex could inhibit breast cancer stem cells (BCSCs) and enhance the cytotoxicity of paclitaxel, which is involved in overcoming resistance to traditional agents (22, 23). Therefore, we questioned whether cuproptosis had a similar function in regulating the TME. A comprehensive understanding of the characteristics of TME cell infiltration mediated by cuproptosis may provide important insights regarding the underlying mechanisms of BRCA tumorigenesis and predict the response to immunotherapy.

This study integrated the genomic information of BRCA samples from seven public databases to comprehensively evaluate cuproptosis patterns and investigated the relationship of cuproptosis patterns with prognosis and TME cell-infiltrating characteristics. We identified two cuproptosis patterns with distinct prognoses, and their TME characteristics were consistent with the immune-excluded and immune-inflamed phenotypes, respectively. This indicated that cuproptosis plays an important role in mimicking the characteristics of individual TME. Additionally, a scoring system was established to quantify the cuproptosis pattern in individual patients to accurately predict patient outcomes and their response to immunotherapy.

## Materials and methods

### BRCA data source and preprocessing

The training cohort from The Cancer Genome Atlas (TCGA)-BRCA gene expression profiles in the fragments per kilobase million (FPKM) format was downloaded from the TCGA databases using the R package “TCGAbiolinks,” and then log2 transformed for normalization. The simple nucleotide variation (SNV) and copy number variation (CNV) data were acquired from the TCGA database. The survival information (DFI) and clinical data of patients, which were adjusted by TCGA official, were obtained from the work of Liu et al., and 907 tumor samples with both expression and survival data were retained for subsequent analysis (24).

The gene expression profiles of the validation cohort data were downloaded from the Gene-Expression Omnibus (GEO) database, including GSE21653_GPL570, GSE7390_GPL96, GSE42568_GPL570, GSE11121_GPL96, GSE12093_GPL96, and GSE17705_GPL96. The probes were converted into gene symbols according to the corresponding platform annotation file. If a probe corresponded to multiple genes, the probe was excluded, and if multiple probes corresponded to the same symbol, the median value was calculated. The immunotherapy cohort treated with anti-PD-1 therapy (PRJEB25780) was downloaded from the Tumor Immune Dysfunction and Exclusion database. Another immunotherapy cohort treated with bevacizumab (GSE53127) was downloaded from the GEO database.

### Unsupervised clustering for cuproptosis-related genes

According to the expression profile data of 10 cuproptosis-related genes (CRGs), unsupervised clustering analysis was used to identify distinct cuproptosis patterns and classified patients for further analysis. A consensus clustering algorithm was used to determine the number of clusters and their stability. The R package “ConsensusClusterPlus” was employed for the analysis using optimal km clustering, distance metric Pearson, using 1,000 times-cycle computation to ensure the stability and reliability of classification (“km” and “Pearson” are functions in R). The optimal number of classifications was identified based on the proportion of ambiguously clustered pairs metric (25).

### Gene set variation analysis and functional annotation

The differences in the biological processes of a number of cuproptosis patterns were ascertained by GSVA using the “GSVA” package in R. GSVA is a non-parametric and unsupervised method commonly used to estimate changes in pathways and biological process activity in the samples of an expression dataset. We downloaded the HALLMARK gene set “msigdb.v7.4.symbols.gmt” from the MSigDB database (https://www.gsea-msigdb.org/gsea/index.jsp) to run the GSVA analysis. Differences were considered statistically significant at adjusted P values< 0.05. The “clusterProfiler” R package was used to perform functional annotation for CRGs, with the parameters: pvalueCutoff = 0.05, and pAdjustMethod = “BH”.

### Estimation of TME cell infiltration

We calculated the proportion of the immune cell infiltration in the BRCA TME based on the following three methods and compared the distribution of the immune cell infiltration in different groups of samples using the Wilcoxon singed-rank test.

The relative abundance of each cell infiltration in the BRCA TME was determined by a single-sample gene set enrichment analysis (ssGSEA) algorithm. The gene sets of TME infiltration for each immune cell type were selected from the work of Charoentong et al., which included 28 human immune cell types (26). We calculated enrichment scores using ssGSEA analysis to represent the relative abundance of each TME-infiltrating cell in each sample.

CIBERSORT algorithm was used in combination with signature matrix LM22 to estimate the proportion of 22 immune cell phenotypes in each BRCA sample, with the sum of the proportion of all estimated immune cell types in each sample equal to 1.

The xCell algorithm was used to calculate the infiltration proportion of 64 kinds of immune cells using “IOBR” package in R.

### Evaluation of TME Scores

The immune scores, stromal scores, and tumor purity of each BRCA sample were calculated by the “ESTIMATE” package in R. Wilcoxon tests were then used to compare the differences in the scores between groups.

### Differentially expressed genes among cuproptosis patterns

DEGs between distinct cuproptosis patterns were determined by “limma” R package. The significance criteria for determining DEGs was set as |log2FC|≧1 and FDR<0.01.

### Construction of cuproptosis score

We constructed a scoring system to quantify the cuproptosis patterns in individual tumors of BRCA patients. The procedures for establishment of cuproptosis score were as follows: univariate Cox regression analysis was used to determine the hazard ratio (HR) and prognostic significance of DEGs between cuproptosis patterns and the prognostic genes were set to P value< 0.05. Finally, we performed principal component analysis (PCA) based on prognostic genes to construct cuproptosis-related gene signature. Both principal components 1 and 2 were selected to act as signature scores. The advantage of this approach was to concentrate the score on the genes with the largest block of well correlated (or anticorrelation) in the gene set, while reducing contributions from genes that did not track with other set members. The formula is as follow:


$$Score\;=\;\sum \text{ (}PC1_{i}+\;PC2_{i})$$

where i is the expression of cuproptosis phenotype-related genes.

The samples were divided into high and low groups according to the median value of cuproptosis score, and the correlation between these two types of samples and disease-free interval (DFI) was further analyzed.

### Prediction of drug sensitivity

Based on the genomics of drug sensitivity in cancer (GDSC) v2 (https://www.cancerrxgene.org/) database, using the calcPhenotype algorithm in “oncoPredict” R package to evaluate the drug IC50 value of each sample in the training set.

### Gene set enrichment analysis and functional annotation

Gene set enrichment analysis (GSEA) was performed between high and low cuproptosis score groups by the “clusterProfiler” package in R (27). The R package “enrichplot” was used for visualization of the GSEA results.

### Statistical analysis

All data analyses were performed using R Version 4.1.2. When conducting significance analysis among various values, such as expression, infiltration proportion, various characteristic values, Wilcoxon singed-rank and Kruskal-Wallis tests were performed to compare differences between two and multiple groups of samples, respectively. In the figures, the asterisks indicate the statistical P value (ns, P ≥ 0.05, *P< 0.05, ** P< 0.01, *** P< 0.001, and **** P< 0.0001). The PAM50 genotypes (Basal, Her2, LumA, LumB, and Normal) of BRCA patients were determined by PAM50 function that provided by R package “genefu” (28). Survival curves for prognostic analysis were generated using the Kaplan-Meier method, and the significance of the differences was determined using log-rank tests. The CNV landscape of 10 CRGs in the 23 pairs of chromosomes was plotted using the R package “RCircos”. The R package “maftools” was used to present the mutation landscape of the CRGs (29).

## Results

### Genetic and transcriptional alterations of the cuproptosis-related genes in BRCA

**Figure 1:**
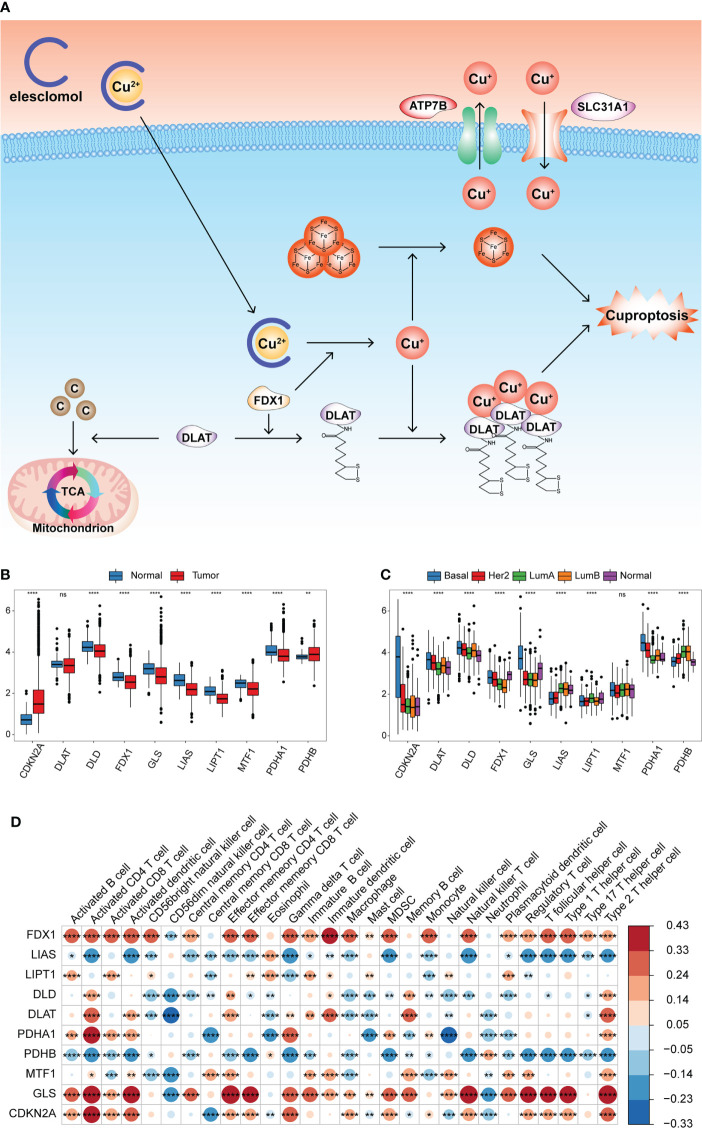
Transcriptional alterations of the CRGs and their relationships with TME cell infiltration in BRCA. **(A)** Summary of cuproptosis mechanism. Elesclomol is a copper ionophore to bind Cu^2+^ and transport it into cells. FDX1 reduces Cu^2+^ to Cu^+^ and promotes the lipoylation of enzymes (especially DLAT) involved in the regulation of mitochondrial TCA cycle. Cu^+^ promotes lipoylated protein aggregation and iron-sulfur cluster protein loss, which triggered proteotoxic stress and cell death. Copper importers (e.g., SLC31A1) and exporters (e.g., ATP7B) affected cuproptosis sensitivity by regulating intracellular copper levels. **(B)** Boxplot shows the expression of 10 CRGs between tumor and normal tissues in the TCGA-BRCA cohort. Normal, blue; Tumor, red. **(C)** Boxplot shows the expression of 10 CRGs among different PAM50 phenotypes. **(D)** Heatmap of correlation between CRGs expression and the enrichment scores of TME infiltrating cells. (^ns^,*P* ≥ 0.05, ^*^ *P* < 0.05, ^**^ *P* < 0.01, ^***^ *P* < 0.001, and ^****^ *P* < 0.0001).

Latest research has identified 10 genes to be responsible for copper-induced cell death through genome-wide CRISPR-Cas9 loss-of-function screens and individual gene knockout studies. Seven genes (FDX1, LIAS, LIPT1, DLD, DLAT, PDHA1, and PDHB) played a positive role in cuproptosis, whereas the other three genes (MTF1, GLS, and CDKN2A) played a negative role. **Figure 1A** summarizes the mechanism of cuproptosis. The workflow of this study is shown in **Figure S1A**. We first evaluated transcriptional alterations in the 10 CRGs between normal and BRCA tissues. The expression of the seven CRGs was lower in BRCA tissues than in normal breast tissues, whereas only the expression of CDKN2A was significantly higher in BRCA tissues (**Figure 1B**). Further analysis showed that nine genes were also significantly differentially expressed in different PAM50 genotypes, whereas only a small number of genes were differentially expressed in other clinical characteristic groups (age, stage, and menopause status) (**Figure 1C** and **S1B-D**). To ascertain whether genomic variations were responsible for these mRNA expression differences, we assessed the incidence of CNVs and somatic mutations in tumor samples from the TCGA dataset; we found none of the 10 CRGs to be mutated, and no CNV was discovered (**Figure S1E**). The locations of the 10 CRGs on the chromosomes are shown in **Figure S1F**. The above analyses demonstrated that the expression pattern of the CRGs was highly heterogeneous between normal and tumor tissues and among different PAM50 phenotypes, indicating that the expression imbalance of the CRGs may be caused by abnormal transcription and may play a vital role in BRCA initiation and progression.

This study aimed to explore the prognostic value of these CRGs in BRCA. According to the expression median value of each CRG, the samples were divided into high and low expression groups, and the effect of CRG expression on survival was evaluated using univariate Cox regression analysis. Patients with low PDHA1 expression had longer overall survival (OS) than those with high PDHA1 expression (P< 0.05, **Figure S2A, B**). Further, we comprehensively explored the functional roles of the CRGs in the TME. The ssGSEA algorithm was run using the expression profile data of tumor samples in the TCGA-BRCA cohort, and the enrichment scores of 28 types of immune cells were obtained. The Pearson correlation analysis was performed to calculate the correlation between the expression of the CRGs and the enrichment scores. The expression of FDX1 and GLS was significantly positively correlated with most of the enrichment scores, whereas the expression of LIAS and PDHB was significantly negatively correlated with most of the enrichment scores (**Figure 1D** and **Table S1**). This indicated that the expression of the CRGs was significantly correlated with the levels of immune cell infiltration in the BRCA TME, which may play a crucial role in prognosis and response to immunotherapy.

### Identification of cuproptosis patterns in BRCA

**Figure 2:**
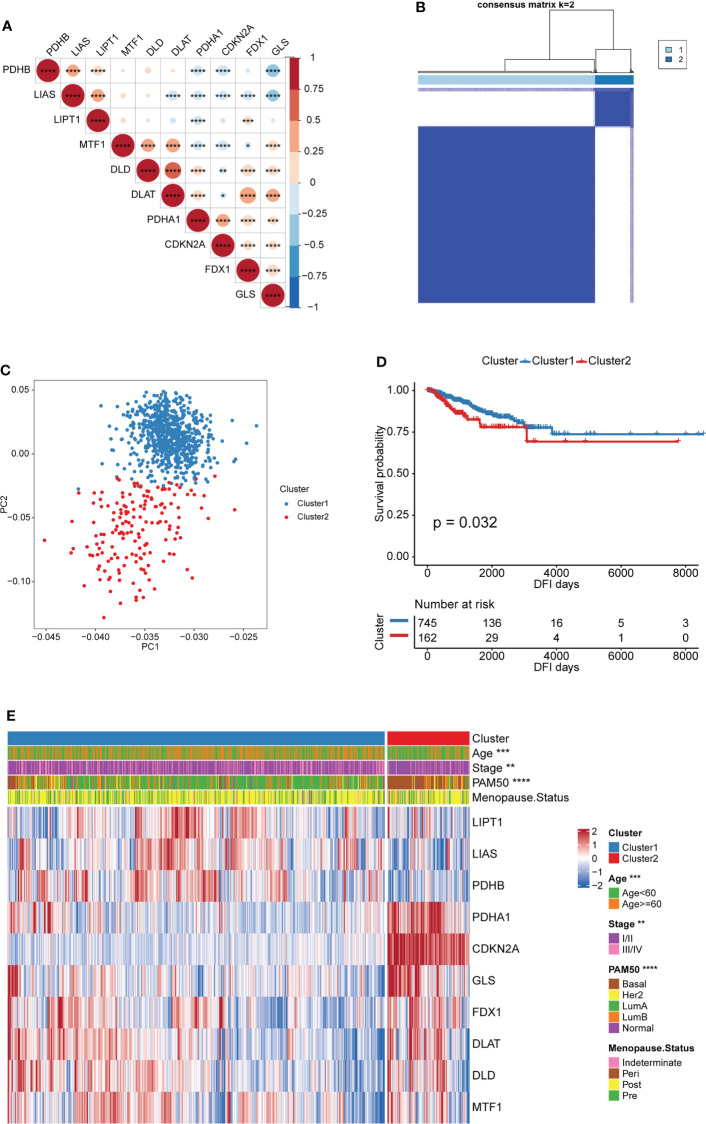
Patterns of cuproptosis and clinical characteristics of each pattern. **(A)** Expression correlation of CRGs. Negative correlation: blue; positive correlation: red. **(B)** Consensus clustering of the CRGs matrix for k = 2 of 907 patients in the TCGA-BRCA cohort. **(C)** Principal component analysis for the transcriptome profiles of two cuproptosis patterns, indicating a remarkable difference on transcriptome between different cuproptosis patterns. **(D)** Kaplan–Meier curves for DFI of TCGA-BRCA cohort with the cuproptosis patterns. **(E)** Unsupervised clustering of 10 CRGs in the TCGA-BRCA cohort. The cuproptosis patters, age, stage, PAM50 subtypes and menopause status were used as sample annotations. Red represents high expression of CRGs, and blue represents low expression. (^*^ *P* < 0.05, ^**^ *P* < 0.01, ^***^ *P* < 0.001, and ^****^ *P* < 0.0001).

The Spearman correlation analysis was performed among the 10 CRGs, and significant correlation was found among most of them (**Figure 2A** and **Table S2**). This suggested that the 10 CRGs may have constructed complex networks to integrally regulate cuproptosis; these networks may be involved in the formation of distinct cuproptosis patterns and the TME-cell infiltrating characteristics of individual tumors to influence BRCA development.

Based on the expression of the 10 CRGs, two distinct cuproptosis patterns were identified via an unsupervised clustering algorithm of the R package “ConsensusClusterPlus”. These were called Cluster1 and Cluster2 and included 745 and 162 cases, respectively (**Figure 2B** and **Table S3**). In addition, principal component analysis (PCA) revealed remarkable differences between the cuproptosis transcription profiles of the two patterns, suggesting that the unsupervised clustering was successful (**Figure 2C**). The Kaplan-Meier curves showed a longer DFI in patients from Cluster1 than in those from Cluster2 (**Figure 2D**). Furthermore, the relationship between the two patterns and the BRCA clinical characteristics was studied. As shown in **Figure 2E**, patients with LumA and Basal subtypes were characterized by Cluster1 and Cluster2, respectively. The Basal subtype was significantly associated with the worst prognosis in BRCA, whereas the LumA subtype was associated with the best clinical outcomes. Moreover, the expression pattern of the CRGs showed a difference between the distinct cuproptosis patterns. Cluster1 was characterized by the increased expression of most cuproptosis-promoting genes (LIPT1, LIAS, PDHB, FDX1, DLAT, and DLD), whereas Cluster2 was characterized by the increased expression of two cuproptosis-inhibiting genes (CDKN2A and GLS) and a cuproptosis-promoting gene (PDHA1) (**Figure 2E**). This indirectly suggested that cuproptosis may inhibit BRCA progression by inducing tumor cell death.

**Figure 3:**
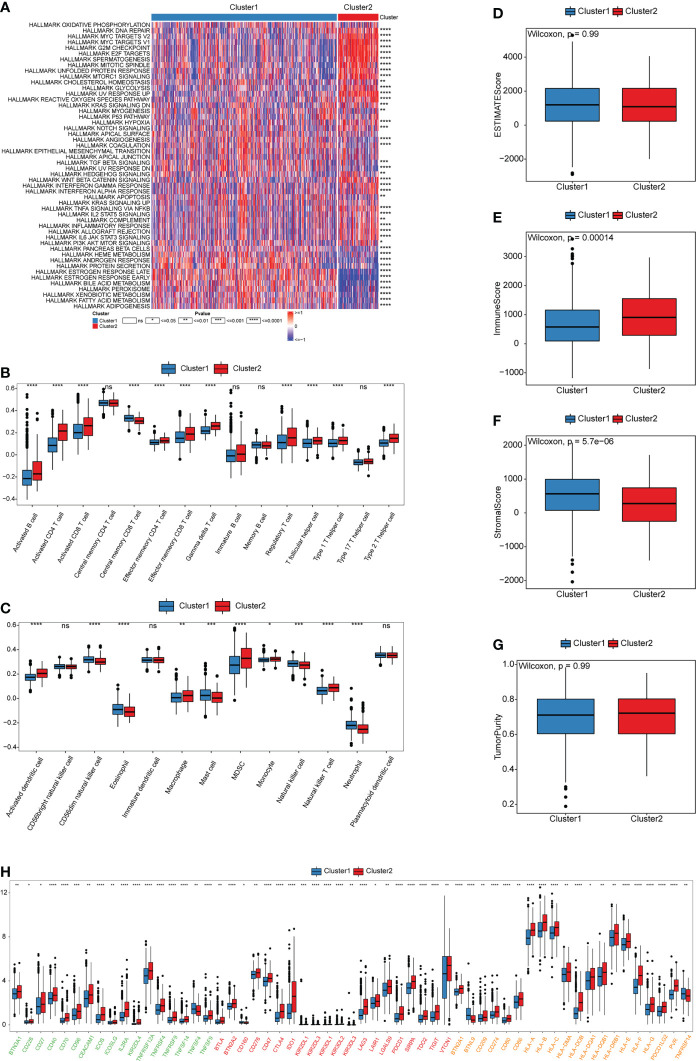
Biological and TME characteristics of cuproptosis patterns. **(A)** GSVA enrichment analysis demonstrates the activation states of HALLMARK pathways between distinct cuproptosis patterns in TCGA-BRCA cohort and visualized by heatmap. Red and blue represent activated and inhibited pathways, respectively. The cuproptosis patterns were used as sample annotations. **(B, C)** The infiltrating abundance of 28 immune cell types in two cuproptosis patterns. **(B)** represents adaptive immunity; **(C)** represents innate immunity. **(D-G)** Boxplot shows the difference of ESTIMATE score, immune score, stromal score, and tumor purity between cuproptosis patterns. **(H)** Expression of ICB-related genes in two cuproptosis patterns. Green, activate; Red, inhibit; Orange, two-side. (^ns^,*P* ≥ 0.05, ^*^ *P* < 0.05, ^**^ *P* < 0.01, ^***^ *P* < 0.001, and ^****^ *P* < 0.0001).

GSVA enrichment analysis was performed to identify biological behavioral differences between the two cuproptosis patterns. Most of the 50 pathways in the HALLMARK data set had significant differences in enrichment between the two patterns. Cluster1 was significantly enriched in the estrogen response early and late pathways and the stromal activation-related TGF β pathways. However, Cluster2 was significantly enriched in tumor immune escape- and carcinogenesis-related pathways, such as the glycolysis, ROS, mTORC1, Hedgehog, Wnt/β-catenin, Myc, and inflammatory response pathways (**Figure 3A** and **Table S4**). These analyses suggest that the cuproptosis pattern was closely related to the biological behavior of tumors in BRCA.

To investigate the role of cuproptosis in the TME of BRCA, we evaluated the enrichment scores of the 28 types of immune cells in the two cuproptosis patterns using ssGSEA analysis. Subsequently, we evaluated the tumor purity and TME score (stromal, immune, and estimate scores) of the two patterns using the “ESTIMATE” package. We observed significant differences in the infiltration of most immune cells between the two groups. Cluster1 was markedly abundant in innate immune cell infiltration, such as CD56dim natural killer (NK) cells, eosinophils, mast cells, NK cells, and neutrophils (**Figure 3B** and **Table S5**). However, there were no significant differences in tumor purity and ESTIMATE scores between the two patterns (**Figure 3D, G** and **Table S6**). Cluster1 had a higher stromal score, which indicates a higher relative content of stromal cells in the TME, suggesting that Cluster1 could be considered non-inflamed tumors (**Figure 3E**). According to previous studies, non-inflamed tumors contain immune-excluded and immune-desert phenotypes. The immune-excluded phenotype is characterized by the presence of a large number of immune cells. However, these immune cells cannot penetrate the tumor parenchyma but remain in the stroma around the tumor cell nests. After immunotherapy, stromal-associated T cells can show activation and proliferation, but their infiltration is blocked by the stroma and shows no response to immunotherapy (30). Contrarily, inflamed tumors exhibit an immune-inflamed phenotype, which is characterized by the presence of both CD4- and CD8-expressing T cells in the tumor parenchyma, usually accompanied by myeloid cells and monocyte cells, and the immune cells are located near the tumor cells. Importantly, the response to anti-PD-L1/PD1 therapy most frequently occurs in patients with inflamed tumor (31–33). Patients from Cluster2 exhibited higher immune scores and a higher infiltration of most adaptive immune cells, especially activated CD4 and CD8 T cells, and dendritic cells (DCs) (**Figure 3C, F**). Therefore, patients from Cluster2 could be recognized as having the immune-inflamed phenotype, which is exhibited by inflamed tumors. DCs are responsible for antigen presentation and the activation of naive T cells, which is a bridge between innate and adaptive immunity, and the activation of which depends on high expression levels of MHC molecules, co-stimulators, and adhesion factors (34, 35). We estimated the expression of ICB-related genes among the two patterns. We observed that the expression of PDCD (PD1), CD274 (PD-L1), and MHC molecules (including HLA-A, HLA-B, HLA-C, HLA-DMA, HLA-DOB, HLA-DQA1, HLA-DRB1, HLA-E, HLA-F, and HLA-G) was higher in Cluster2 than in Cluster1 (**Figure 3H**). This suggested that patients from Cluster2 might be more suitable for anti-PD-L1/PD1 therapy than those from Cluster1. In addition, the proportion of infiltrating immune cells was evaluated using the CIBERSORT and xCell algorithms, which were highly consistent with previous results of ssGSEA analysis (**Figures S3A, B** and **Tables S7-8**).

### Construction of cuproptosis signatures and functional annotation

**Figure 4:**
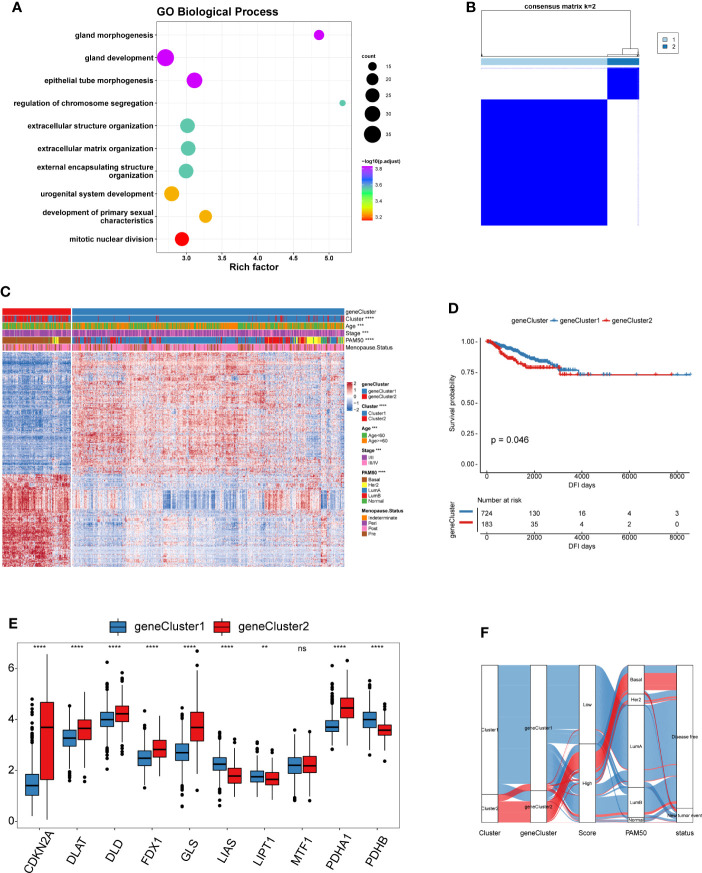
Landscape of biological characteristics of cuproptosis genomic subtypes. **(A)** GO enrichment analysis for cuproptosis phenotype-related DEGs. The x-axis indicates the rich factor within each GO term. **(B)** Consensus clustering of the 601 cuproptosis phenotype-related DEGs for k = 2 of 907 patients in the TCGA-BRCA cohort. **(C)** Unsupervised clustering of 601 cuproptosis phenotype-related DEGs to classify patients into different genomic subtypes, termed as geneCluster1 and geneCluster2, respectively. The gene clusters, cuproptosis patterns, age, stage, PAM50 phenotypes, and menopause status were used as patient annotations. **(D)** The survival curves of different gene clusters in the TCGA-BRCA cohorts were estimated by the Kaplan–Meier plotter. **(E)** Boxplot shows the expression of 10 CRGs between two gene clusters. geneCluster1, blue; geneCluster2, red. **(F)** Alluvial diagram showing the changes of cuproptosis patterns, gene clusters and disease progression status. (^ns^,*P* ≥ 0.05, ^**^ *P* < 0.01, ^***^ *P* < 0.001, and ^****^ *P* < 0.0001).

To gain a further understanding of the potential biological features of each cuproptosis pattern, we identified 601 cuproptosis phenotype-related differentially expressed genes (DEGs) (**Table S9**). Gene Ontology (GO) enrichment analysis of these DEGs showed that their functions were mainly enriched in biological processes that are significantly related to epithelial tube morphogenesis and extracellular matrix organization, which have been demonstrated to shape the TME (**Figure 4A** and **Table S10**) (36–38). Moreover, the molecular function and cellular component enrich analysis showed that the DEGs were also closely related to the collagen-containing extracellular matrix and tubulin binding functions (**Figures S3C, D**). These results further confirmed that the CRGs play a non-negligible role in the TME of BRCA. To further verify this mechanism, we performed unsupervised clustering analysis based on 601 DEGs to divide the tumor samples into different genomic subtypes (**Figure 4B**). The results were similar to the clustering grouping of the cuproptosis patterns, i.e., two distinct genomic phenotypes, designated geneCluster1 and geneCluster2 (**Figure 4C**). The survival analyses showed that the patients with geneCluster1 had better DFI than those with geneCluster2 (**Figure 4D**). Moreover, geneCluster2 was associated with younger age. Similar to the cuproptosis patterns, patients with LumA and Basal subtypes were characterized by the geneCluster1 and geneCluster2, respectively. Unsurprisingly, there were significant differences in the expression of the nine CRGs between the two gene clusters. (**Figure 4E**).

### Construction of a scoring system for quantifying the cuproptosis patterns of individual patients with BRCA

**Figure 5:**
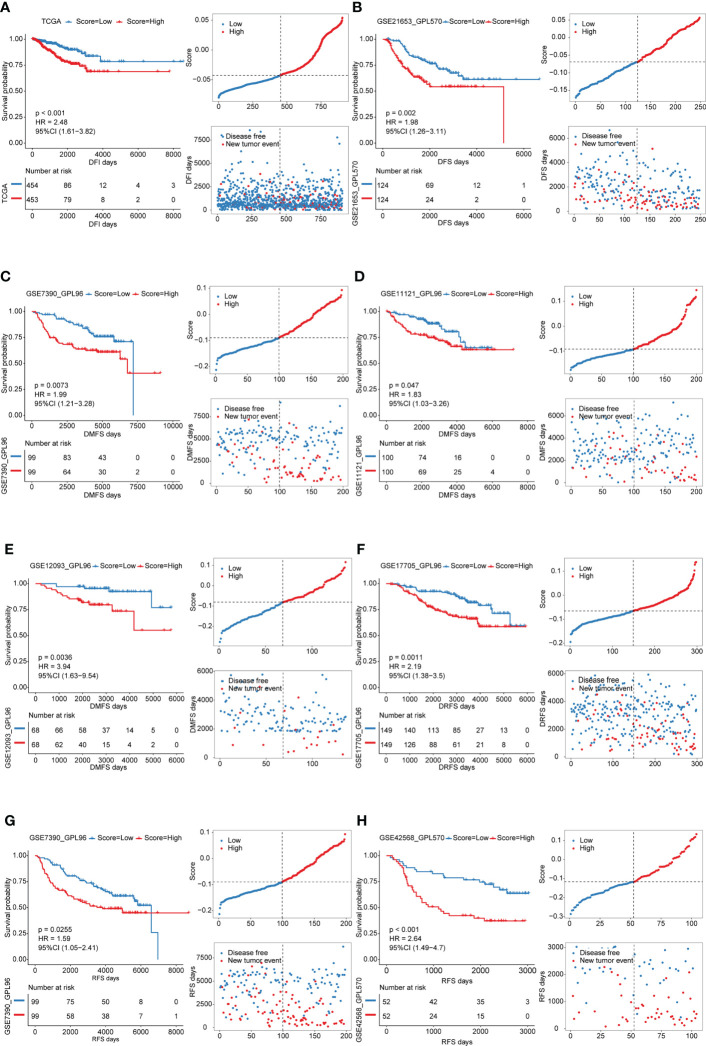
Survival analyses for low and high cuproptosis score groups in the training cohort and validation cohorts. Distribution of patients in the training cohort and validation cohorts based on the median risk score and recurrence status of each patient. **(A)** training cohort; **B-H** validation cohorts.

**Figure 6:**
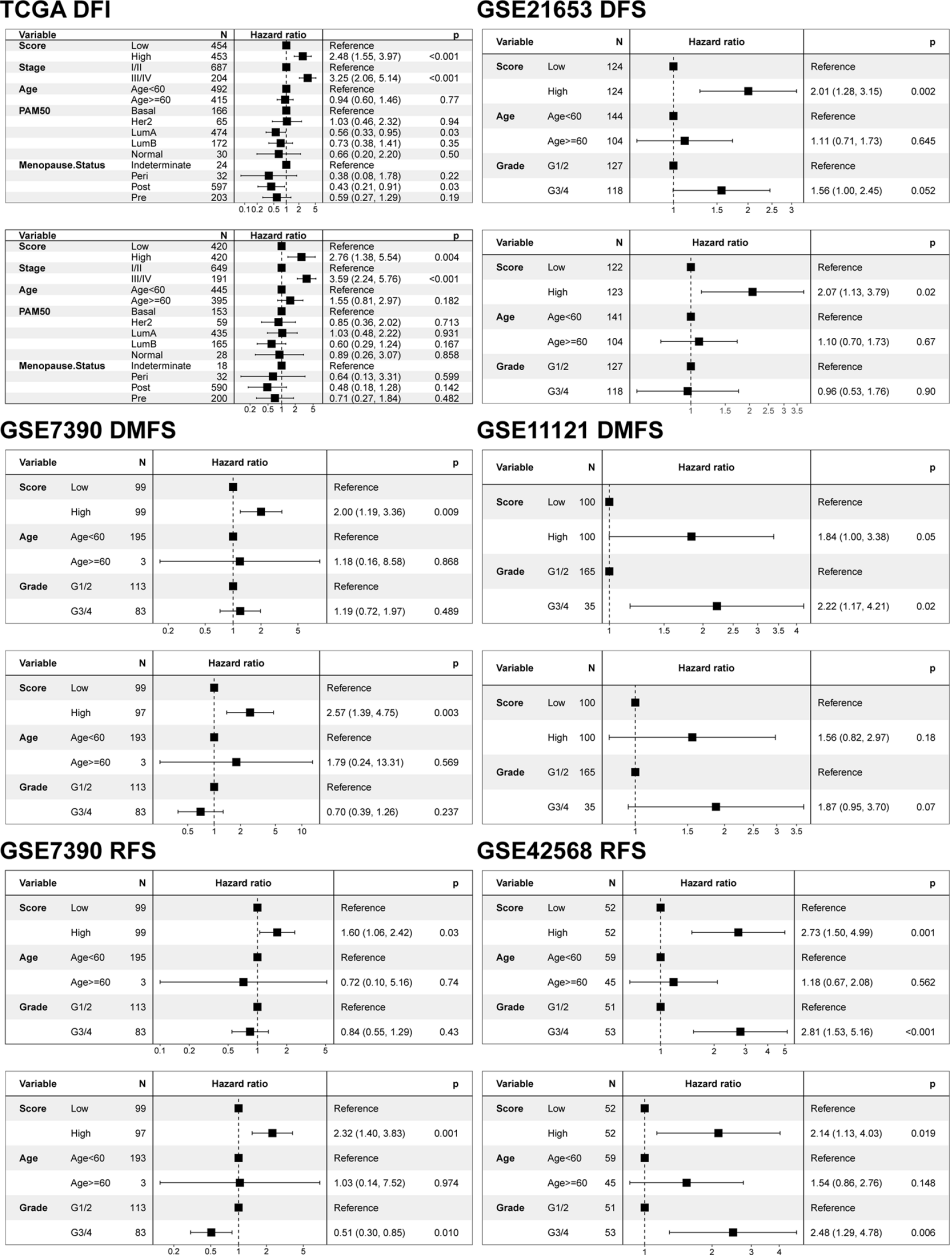
Univariate and multivariate Cox regression model analysis of the factors including cuproptosis score, age, stage, grade, PAM50 genotypes, and menopause status in the training and validation cohorts.

In view of the heterogeneity and complexity of individual cuproptosis patterns, we constructed a scoring system based on cuproptosis phenotype-related DEGs to accurately predict cuproptosis patterns in individual patients. Univariate Cox analysis was performed on the cuproptosis phenotype-related DEGs obtained from the previous analysis and 120 genes related to prognosis were screened (**Table S11**). The cuproptosis score was calculated using PCA. According to the median value of the cuproptosis score (-0.04260481), the samples were divided into high and low cuproptosis score groups. The results showed that patients with a low cuproptosis score had better DFI than patients with a high cuproptosis score, indicating that the cuproptosis score could characterize and predict the DFI of patients with BRCA well (**Figure 5A**). Based on these 120 prognostic genes, the performance of the cuproptosis scoring system was validated using the same method in six additional validation cohorts. The results were consistent and showed that patients in the low cuproptosis score group experienced better disease-free survival (DFS), distant metastasis-free survival (DMFS), distant relapse-free survival (DRFS), and recurrence-free survival (RFS) relative to that in the high cuproptosis score group (**Figures 5B-H**). Finally, receiver operating characteristic (ROC) curves were constructed based on the cuproptosis score to summarize the predictive ability (**Figure S4**). Furthermore, we performed univariate and multivariate Cox regression analyses using patient clinical characteristics, including age, stage, grade, PAM50 genotypes, and menopause status. We demonstrated that cuproptosis score was a robust and independent prognostic biomarker for evaluating outcomes in patients of the TCGA-BRCA training cohorts and the validation cohorts with clinical characteristics (**Figure 6**). However, in the GSE11121 validation cohort, the cuproptosis score was not an independent prognostic biomarker, which may be explained by the large difference in the data distribution of the sample grade in this dataset. An alluvial diagram was used to better visualize the distribution of BRCA samples among the different cuproptosis patterns, genomic subtypes, PAM50 genotypes, and cuproptosis score groups (**Figure 4F**).

**Figure 7:**
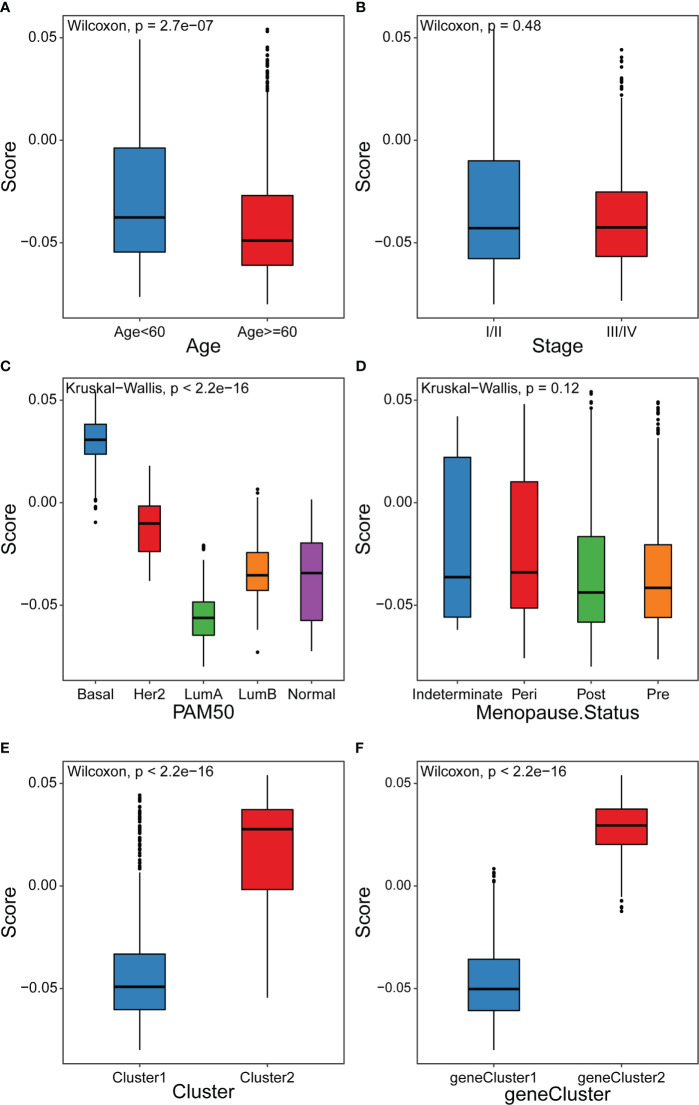
Distribution of cuproptosis score in different groups **(A-D)** Differences in cuproptosis score among distinct clinical features related subgroups in the TCGA-BRCA cohort. **(E)** Differences in cuproptosis score among two cuproptosis patterns in TCGA-BRCA cohort **(F)** Differences in cuproptosis score among two gene clusters in TCGA-BRCA cohort.

Our analyses have revealed survival prognostic differences between the high and low cuproptosis score groups. Furthermore, we explored the latent mechanism behind these results. We analyzed the relationship between the cuproptosis score and clinical characteristics, cuproptosis patterns, and genomic phenotypes in the TCGA-BRCA cohort. As shown in **Figures 7A-D**, the cuproptosis score was higher in the groups with younger ages and Basal subtype. Previous studies have demonstrated that younger patients and those with basal subtype BRCA have a poorer prognosis, which is consistent with the results that patients with low cuproptosis scores had better DFI (39). These results elucidated the fact that patients with a high cuproptosis score had a worse survival prognosis. More importantly, Cluster1 showed a lower cuproptosis score compared to Cluster2, suggesting that patients with a higher cuproptosis score may be related to inflamed tumors (**Figure 7E**). In addition, geneCluster1 showed significantly decreased cuproptosis score compared to geneCluster2, with these patients having a better prognosis (**Figure 7F**). The above results suggested that the cuproptosis score could better evaluate the cuproptosis pattern and TME cell-infiltration of individual patients.

### Molecular mechanism of cuproptosis score

**Figure 8:**
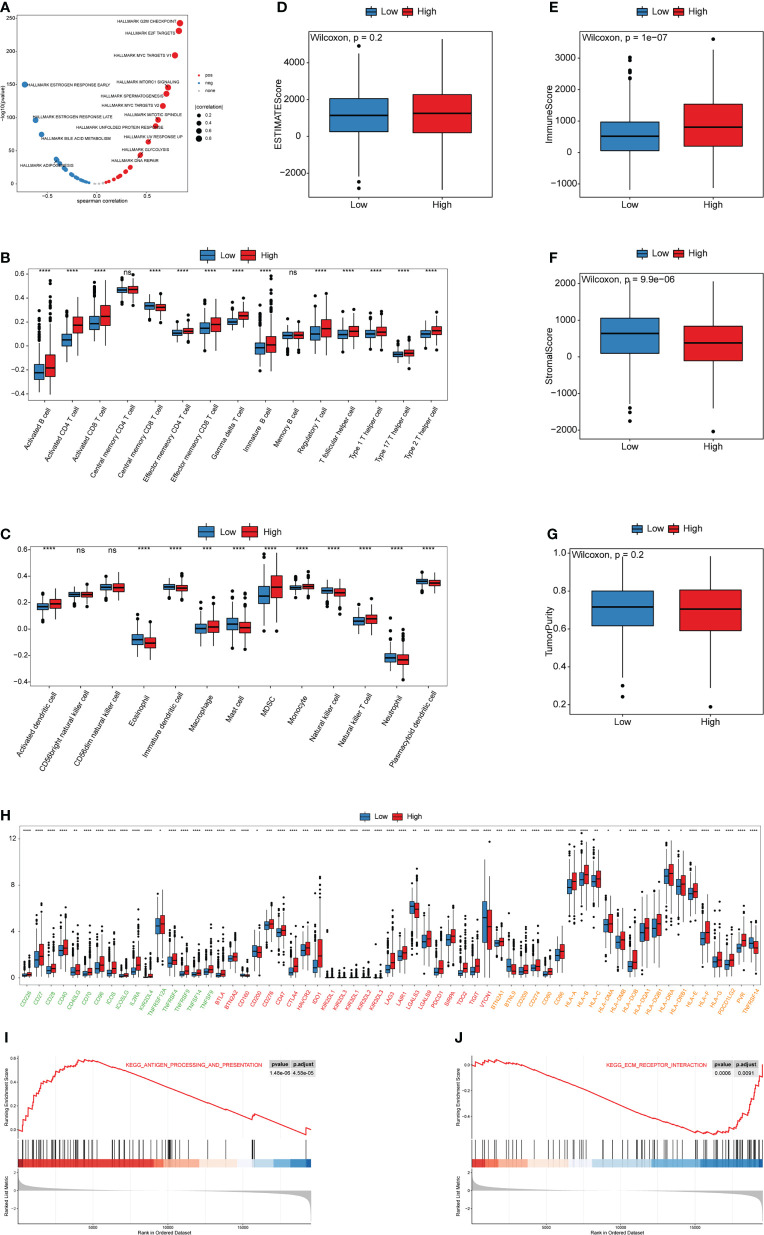
Biological and TME characteristics of distinct cuproptosis score groups **(A)** Spearman correlation analysis between cuproptosis score and HALLMARK pathway activity. **(B, C)** The infiltrating abundance of 28 immune cell types in two cuproptosis score groups. **(B)** represents adaptive immunity; **(C)** represents innate immunity. **(D-G)** Boxplot shows the difference of ESTIMATE score, immune score, stromal score, and tumor purity between cuproptosis score groups. **(H)** Expression of ICB-related genes in two cuproptosis score groups. Green, activate; Red, inhibit; Orange, twoside. **(I, J)** The GSEA results for distinct cuproptosis score groups. (^ns^,*P* ≥ 0.05, ^*^ *P* < 0.05, ^**^ *P* < 0.01, ^***^ *P* < 0.001, and ^****^ *P* < 0.0001).

To further explore the relationship of the cuproptosis score with biological functions and TME cell-infiltration, Spearman correlation analysis between the cuproptosis score and HALLMARK pathways activity was calculated. The results showed that the cuproptosis score was significantly correlated with most pathway activities (**Table S12**). The low cuproptosis score was correlated with estrogen response pathways, which were consistent with the Cluster1 cuproptosis pattern, whereas the high cuproptosis score was associated with the Myc, mTORC1, and glycolysis pathways, which were similar to Cluster2 (**Figure 8A**). Furthermore, we sought to identify the value of cuproptosis in evaluating TME cell-infiltration, while the infiltration of 28 types of immune cells and TME score were further studied in the high and low cuproptosis score groups. Surprisingly, differences in the TME cell-infiltration and TME score between groups with high and low cuproptosis scores were consistent with the results of the two cuproptosis patterns (**Figures 8B-G**). In the low cuproptosis score group, the infiltration of innate immune cells and stromal score were higher, suggesting that the low cuproptosis score group could be considered as non-inflamed tumors. In contrast, the high cuproptosis score group showed higher infiltration of most types of immune cells and a higher immune score, indicating that the high cuproptosis score group could be recognized as inflamed tumors. Moreover, from the results of the expression of ICB-related genes among the low and high cuproptosis score groups it was noted that patients in the high cuproptosis score group exhibited higher PDCD1, CD274, and MHC molecules. This indicates that patients in the high cuproptosis score group might be more sensitive to anti-PD-L1/PD1 therapy (**Figure 8H**). In addition, the proportion of infiltrating immune cells was evaluated using the CIBERSORT and xCell algorithms (**Figures S5A-B**). GSEA results showed that the high cuproptosis score group was significantly enriched in the antigen processing and presentation pathway, whereas the low cuproptosis score group was substantially enriched in the extracellular matrix (ECM) receptor interaction pathway (**Figures 8I, J**, **S6** and **Table S13**). Hence, these results suggest that the cuproptosis score is closely correlated with TME cell infiltration and the immune therapy response.

**Figure 9:**
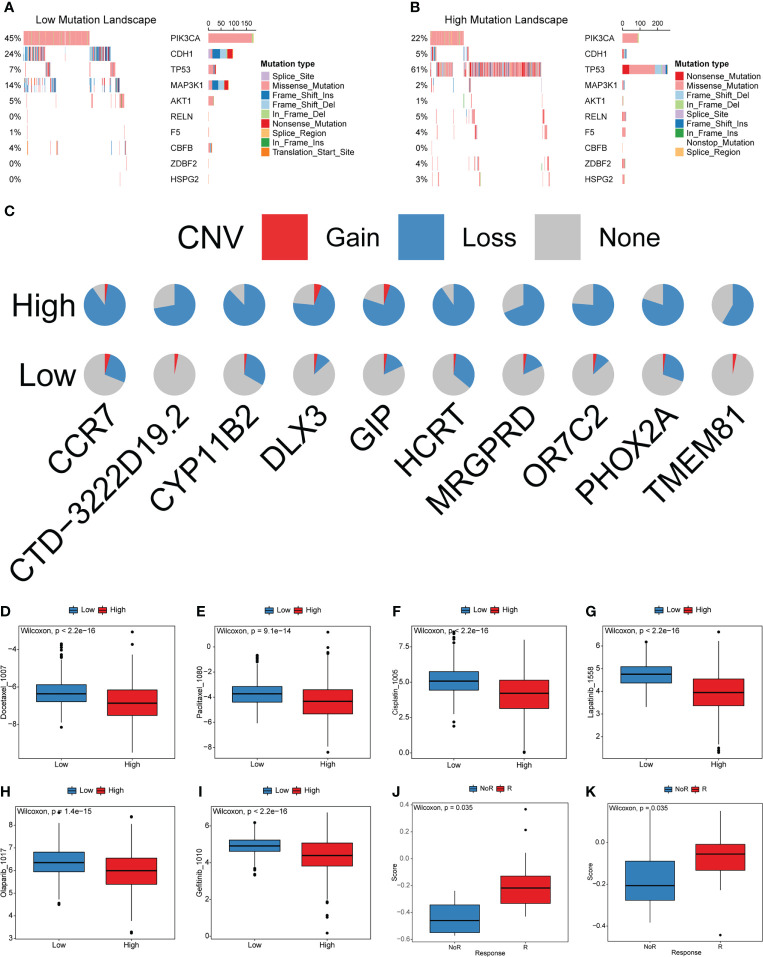
Characteristics of cuproptosis in the mutation landscape and drug response. **(A, B)** The waterfall plot of tumor somatic mutation established by those with low and high cuproptosis score. **(C)** CNV differences in high and low cuproptosis score. **(D-I)** Relationships between cuproptosis score and chemotherapeutic sensitivity. **(J, K)** Cuproptosis score differences between non-response and response immunotherapy groups.

Furthermore, we explored the genetic alterations between the high- and low-cuproptosis score groups. In the TCGA-BRCA training set, Fisher’s exact test was performed to analyze the distribution differences of somatic mutation and CNV between the low and high cuproptosis score groups to explore the genetic alterations between the two groups, and the top 10 were displayed according to the ascending order of p-value (**Figures 9A, B**). The mutation frequency of PIK3CA in the high cuproptosis score group was significantly lower than that in the low cuproptosis score group, while the mutation frequency of the TP53 gene was significantly higher than that in the low cuproptosis score group. Previous studies have shown that TP53 is the most frequently mutated gene in triple-negative breast cancer (TNBC), is more common in the Basal subtype, and has emerged as a major contributor to the suppression of innate immune signaling and promotion of immune escape (40–43). The proportion of copy number loss in CCR7, GIP, and HCRT in the high cuproptosis score group was significantly higher than that in the low cuproptosis score group (**Figure 9C**). All the above results provide a new perspective for exploring the mechanisms of cuproptosis in tumor progression, shaping of the TME landscape, and roles in ICB therapy.

### Predictive ability of the cuproptosis scoring system in the sensitivity of anti-tumor drugs

Most patients with BRCA require chemotherapy, hormone therapy, or targeted therapy to reduce the risks of recurrence and metastasis. However, not all patients are sensitive to these drugs. Moreover, in recent years, many molecular-targeted drugs have been developed for the treatment of BRCA and achieved good results. The above analysis indicates that cuproptosis is closely related to carcinogenesis-related pathways, TME cell infiltration, and other functional pathways. Therefore, the cuproptosis score has potential value for predicting the response to related drugs in individual patients. To verify this ability, we compared the distribution of drug IC50 in the high- and low-cuproptosis score groups using the R package “oncoPredict” and drug information in the GDSC database combined with the expression profile of the training set. The results showed that the IC50 values of docetaxel, paclitaxel, cisplatin, lapatinib, olaparib, and gefitinib were significantly higher in the low cytotoxicity group. These results imply that this model may serve as a predictor of chemotherapy or targeted therapy responsiveness (**Figures 9D-I** and **Table S14**).

Blocking the PD-L1/PD1 pathways is the most representative immunotherapy method, which is undoubtedly being considered as a revolutionary breakthrough in cancer treatment. However, the benefits of ICB treatment remain limited because of innate or acquired resistance to immunotherapy. Considering that the cuproptosis scores were closely related to TME, tumors can be distinguished into non-inflamed and inflamed subtypes. We investigated the ability of the cuproptosis score to predict patient’s response to ICB therapy based on two immunotherapy cohorts. The cuproptosis score was significantly higher in the responding group than in the nonresponding group (**Figures 9J, K**). This result is consistent with our findings, which demonstrated that the high cuproptosis score group was recognized as an inflamed tumor subtype. The above results indicate that patients with the high cuproptosis score could gain greater benefit from ICB treatment than those with low cuproptosis score.

## Discussion

Cuproptosis, a recently proposed form of copper-dependent regulated cell death that is mainly dependent on mitochondrial respiration, has been strongly implicated in cancer (6). Distinct from oxidative stress-induced cell death (e.g., apoptosis, ferroptosis, and necroptosis), cuproptosis is a form of mitochondrial stress-related cell death caused by the aggregation of lipoylated mitochondrial enzymes and loss of Fe–S cluster proteins. Previous studies have found that monotherapy with copper ionophores or therapy in combination with copper showed strong antitumor function in BRCA and has the ability to overcome drug resistance (17–19, 22, 23). Furthermore, combination treatment exhibited better selectivity (20). Other types of regulated cell death have shown the ability to shape the TME and predict the response to anticancer drugs. Enhanced efficacy is achieved by combining inducers and ICB-related drugs for cancer treatment (17, 18). However, the relationship between BRCA and cuproptosis has not been defined. Therefore, the role of cuproptosis in BRCA phenotyping and TME was thoroughly examined in this study, to achieve a comprehensive understanding of the role of cuproptosis in prognosis and TME infiltration characterization. Ascertaining the role of distinct cuproptosis patterns in TME cell infiltration would improve our understanding of TME antitumor immune responses, as well as guide more effective immunotherapy strategies.

In the present study, we first analyzed the genetic and transcriptional alterations in 10 CRGs in BRCA. Eight genes were significantly downregulated in tumor samples, and only CDKN2A was significantly overexpressed. CRGs were differentially expressed in distinct clinical characteristic groups, especially in different PAM50 genotypes. Furthermore, the expression of CRGs significantly correlated with TME cell infiltration. Next, we analyzed the correlation between the 10 CRGs and found that the expression of almost all of them was significantly correlated. Although only the OS curve of PDHA1 in the high- and low-expression groups was significantly different, these CRGs may interact with each other and play a role in BRCA. These results preliminarily confirm that cuproptosis plays an important role in the prognosis and TME cell-infiltrating characteristics of patients with BRCA.

Based on the 10 CRGs, we revealed two distinct cuproptosis patterns that were identified to have significant differences in prognosis and TME cell infiltration characterization. There were significant differences in prognosis between the two patterns, with Cluster2 having a poorer DFI. On exploring the mechanism causing the prognosis differences in the two cuproptosis patterns, GSVA enrichment analysis found that Cluster1 was enriched in the stomal activation related TGFβ and estrogen response signaling pathways. However, Cluster2 was enriched in immune escape and carcinogenesis-related pathways. The TME cell infiltration was further evaluated in distinct cuproptosis patterns. This is consistent with the results of previous analyses that Cluster1 experienced abundant infiltration of innate immune cells and stroma, corresponding to non-inflammatory tumors and immune-excluded phenotypes (44). Although the immune-excluded phenotype is characterized by a high infiltration of immune cells, these immune cells are unable to recognize and eliminate cancer cells because of the abundant stromal elements preventing their penetration into the tumor parenchyma. In contrast, Cluster2 was characterized by the activation of adaptive immunity and abundant immune cell infiltration, corresponding to inflamed tumors and immune-inflamed phenotypes (30). However, patients from Cluster2 did not have a matching survival advantage compared with those from Cluster1. Given the significant upregulation of ICB-related genes in Cluster2, this cuproptosis pattern may be more influenced by ICB pathways. Therefore, we hypothesized that ICB-mediated immune escape in Cluster2 inhibits the antitumor effect of immune cells. We found that Cluster2 and Cluster1 were characterized by patients with LumA and Basal subtypes, respectively. The Basal subtype was significantly associated with the worst survival outcomes in BRCA, whereas the LumA subtype was associated with better clinical outcomes. Patients with TNBC are more likely to benefit from anti-PD-L1/PD1 therapy than those with other BRCA subtypes because of higher immunogenicity, increased enrichment of tumor-infiltrating lymphocytes (TILs), and higher levels of PD-L1 expression (10, 45–47). Importantly, atezolizumab (a monoclonal antibody targeting PD-L1) was approved by the US Food and Drug Administration in combination with nab-paclitaxel for patients with TNBC whose tumors express PD-L1 (48). Based on the prognosis difference and TME cell-infiltrating characteristics of each cluster, the robust ability to predict prognosis and differentiate immune phenotypes by distinct cuproptosis patterns was confirmed.

Furthermore, we demonstrated that transcriptome differences between the distinct cuproptosis patterns were significantly correlated with immune-related biological pathways. We identified DEGs among the cuproptosis patterns that were recognized as cuproptosis-related signature genes. Based on these genes, two genomic subtypes were identified that were mainly enriched in epithelial tube morphogenesis and ECM organization biological processes and have been demonstrated to shape the TME. The patients with geneCluster1 had better DFI than those with geneCluster2. Similar to the cuproptosis patterns, patients with the LumA and Basal subtypes were characterized by the geneCluster1 and geneCluster2, respectively. This further proves that cuproptosis plays an important role in BRCA progression and regulation of TME.

Thus, a comprehensive and accurate assessment of cuproptosis patterns would help predict the prognosis and enhance our understanding of the characteristics of TME cell infiltration. There is an urgent need to quantify cuproptosis patterns in individual patients, owing to the individual heterogeneity of cuproptosis. We constructed a cuproptosis scoring system to quantify the cuproptosis patterns in individual patients with BRCA. First, we tested the ability of the cuproptosis score to predict the prognosis of patients with BRCA. Patients with a low cuproptosis score had better survival than those with a high score in the training cohort, which was further validated in six additional validation cohorts. Univariate and multivariate Cox regression analyses showed that the cuproptosis score was an independent prognostic factor of BRCA. Moreover, the basal subtype showed the highest cuproptosis score, whereas LumA exhibited the lowest score among the PAM50 genotypes. Further research revealed that the cuproptosis score was significantly correlated with the most pathway activities. Cluster1 showed a lower cuproptosis score compared to Cluster2, and geneCluster1 also showed a significantly decreased cuproptosis score compared to geneCluster2. The cuproptosis pattern (Cluster1) characterized by an immune-excluded phenotype, showed a lower cuproptosis score, whereas the cuproptosis pattern (Cluster2) characterized by an immune-inflammatory phenotype, exhibited a higher cuproptosis score. We concluded that the cuproptosis score is a reliable and robust measure for the comprehensive evaluation of individual cuproptosis pattern and can be used to further determine the prognosis and tumor immune phenotypes. Patients from the high cuproptosis score group exhibited higher levels of PDCD1, CD274, and MHC molecules, which indicated higher sensitivity to anti-PD-L1/PD1 therapy. This was further confirmed by GSEA analysis, which showed that the high cuproptosis score group was significantly enriched in antigen processing and presentation pathways. Somatic mutations and CNV between the two groups were explored. The mutation frequency of the TP53 gene was significantly higher in the high cuproptosis score group than that in the low cuproptosis score group. Previous studies have shown that TP53 is the most frequently mutated gene in Basal subtype BRCA and has been associated with the suppression of innate immune signaling and the promotion of immune escape (40–43).

To determine whether the cuproptosis score could predict the drug response of individual patients, we analyzed the relationship between the IC50 values and cuproptosis score. The high cuproptosis score was correlated with lower IC50 of docetaxel, paclitaxel, cisplatin, lapatinib, olaparib, and gefitinib, suggesting higher sensitivity. This analysis indicated that the cuproptosis score had a significant value in predicting drug response in patients. More importantly, we explored the correlation of the cuproptosis score with immunotherapy response in two immunotherapy cohorts. The cuproptosis score in the responding group was significantly higher than that in the non-responding group. The above results imply that patients with the high cuproptosis score could benefit substantially from immunotherapy. The cuproptosis scoring system was demonstrated to improve the selection of anticancer drugs and predict the response to immunotherapy.

In summary, the cuproptosis score can be used for the comprehensive assessment of cuproptosis patterns, corresponding prognosis, and TME cell-infiltrating characteristics of individual patients to further verify the immune phenotypes of tumors and direct more effective therapeutic schedules. The cuproptosis score may also be used to evaluate patients’ clinicopathological features, including age and PAM50 genotypes. The cuproptosis score can also predict the efficacy of hormone therapy, chemotherapy, and clinical response of patients to anti-PD-L1/PD1 immunotherapy. More importantly, cuproptosis is a newly discovered pattern of cell death, and we demonstrated, for the first time to our knowledge, that it has an important impact on the prognosis and TME of BRCA. We propose that we may be able to change the cuproptosis pattern by targeting CRGs or cuproptosis phenotype-related genes, thereby reversing the disadvantageous TME cell-infiltrating characteristics and improving immunotherapy efficacy, thus helping to develop new combination immunotherapeutic strategies or new immunotherapy drugs. This could also provide a theoretical basis for the development of more individualized BRCA treatment in the future.

Despite conducting multi-pronged and multi-database verification, this study has certain limitations. First, all analyses were conducted on data from public databases. Large-scale prospective and multi-center data are needed to validate the scoring system. In addition, the underlying mechanism between CRGs and TME cell-infiltrating characteristics and immunotherapy efficacy needs to be further explored via vitro and in vivo experiments. However, cuproptosis patterns and the TME cell-infiltration characteristics may change during treatments, such as neoadjuvant chemotherapy, chemotherapy, hormonal therapy, radiotherapy, and targeted therapy. This may limit the personalized evaluations and treatment. Therefore, continuous monitoring of CRGs and immune markers is essential for developing and adjusting treatment regimens.

## Conclusion

This comprehensive analysis demonstrated the regulatory mechanisms of cuproptosis that affect the prognosis and TME of BRCA. Distinct cuproptosis patterns were identified as factors impacting the heterogeneity and complexity of the TME. The cuproptosis score is a powerful tool for evaluating the cuproptosis pattern in individual patients. A comprehensive evaluation of individual tumor cuproptosis patterns will enhance our understanding of the TME cell-infiltrating characteristics and provide new ideas for guiding more personalized immunotherapeutic strategies.

## Data availability statement

The original contributions presented in the study are included in the article/**Supplementary Material**. Further inquiries can be directed to the corresponding authors.

## Ethics statement

Written informed consent was obtained from the individual(s) for the publication of any potentially identifiable images or data included in this article.

## Author contributions

DP and YC conceived the idea, design the study. WL retrieved and analyzed the data, and drafted the manuscript. WL and XZ revised and polished the manuscript. All authors contributed to the article and approved the submitted version.

## Funding

This work was supported by funding from the Project Nn10 of Harbin Medical University Cancer Hospital (Grant Number Nn102017-02) and the National Natural Science Foundation of China (Grant Number 82173235).

## Acknowledgments

The authors would like to thank the reviewers for their helpful comments on this article, as well as research groups for the TCGA, CEO, and Tumor Immune Dysfunction and Exclusion database which provided data for this collection.

## Conflict of interest

The authors declare that the research was conducted in the absence of any commercial or financial relationships that could be construed as a potential conflict of interest.

## Publisher’s note

All claims expressed in this article are solely those of the authors and do not necessarily represent those of their affiliated organizations, or those of the publisher, the editors and the reviewers. Any product that may be evaluated in this article, or claim that may be made by its manufacturer, is not guaranteed or endorsed by the publisher.

## Supplementary material

Supplementary Figure 1 Expression of CRGs in different clinical characteristic subgroups and genetic alterations. **(A)** Overview of this study. **(B)** Boxplot shows the expression of 10 CRGs between different age groups in the TCGA-BRCA cohort. Age< 60, blue; Age ≧60, red. **(C)** Boxplot shows the expression of 10 CRGs between different stage groups in the TCGA-BRCA cohort. Stage I/II, blue; Stage III/IV, red. **(D)** Boxplot shows the expression of 10 CRGs between different menopause groups in the TCGA-BRCA cohort. Indeterminate, blue; Peri, red; Post, green; Pre, orange. **(E)** The mutation frequency of 10 CRGs in patients from TCGA-BRCA cohort. **(F)** The location of 10 CRGs on 23 chromosomes. (^ns^, *P* ≥ 0.05, ^*^ *P*< 0.05, ^**^ *P*< 0.01, ^***^ *P*< 0.001, and ^****^ *P*< 0.0001)

Supplementary Figure 2 Survival analyses for patients with low and high CRGs expression.

Supplementary Figure 3 TME characteristics and biological features of cuproptosis patterns. **(A)** The abundance of each TME infiltrating cell in cuproptosis patterns. **(B)** The proportion of TME infiltration cell in cuproptosis patterns. **(C, D)** Molecular function and cellular component annotation for cuproptosis phenotype-related DEGs using GO enrichment analysis. (^ns^, *P* ≥ 0.05, ^*^ *P*< 0.05, ^**^ *P*< 0.01, ^***^ *P*< 0.001, and ^****^ *P*< 0.0001)

Supplementary Figure 4 The predictive value of cuproptosis score in training and validation cohorts.

Supplementary Figure 5 TME characteristics of high and low cuproptosis score groups. **(A)** The abundance of each TME infiltrating cell in high and low cuproptosis score groups. **(B)** The proportion of TME infiltration cell in high and low cuproptosis score groups. (^ns^, *P* ≥ 0.05, ^*^ *P*< 0.05, ^**^ *P*< 0.01, ^***^ *P*< 0.001, and ^****^ *P*< 0.0001)

Supplementary Figure 6 The GSEA enrichment analysis in terms of the high and low cuproptosis score groups.

## Abbreviations

BCSC, breast cancer stem cell; BRCA, breast cancer; CNV, copy number variation; CRG, cuproptosis-related gene; DC, dendritic cell; DEG, differentially expressed gene; DFI, disease-free interval; DFS, disease-free survival; DMFS, distant metastasis-free survival; DRFS, distant relapse-free survival; DSF, disulfiram; ECM, extracellular matrix; FPKM, fragments per kilobase million; GDSC, genomics of drug sensitivity in cancer; GEO, Gene-Expression Omnibus; GO, Gene Ontology; GSEA, gene set enrichment analysis; GSVA, gene set variation analysis; HR, hazard ratio; ICB, immune checkpoint blockade; NK, natural killer; OS, overall survival; PCA, principal component analysis; RFS, recurrence-free survival; ROC, receiver operating characteristic; SNV, simple nucleotide variation; ssGASE, single-sample gene set enrichment analysis; TCGA, The Cancer Genome Atlas; TIL, tumor-infiltrating lymphocyte; TME, tumor microenvironment; TNBC, triple-negative breast cancer; TNM, tumor-node-metastasis.

## References

[1] Sung H Ferlay J Siegel RL Laversanne M Soerjomataram I Jemal A. Global cancer statistics 2020: GLOBOCAN estimates of incidence and mortality worldwide for 36 cancers in 185 countries. CA Cancer J Clin (2021) 71:209–49. doi: 10.3322/caac.21660 33538338

[2] Waks A Winer EJJ. Breast cancer treatment: A review. Jama (2019) 321:288–300. doi: 10.1001/jama.2018.19323 30667505

[3] Brooks M Burness M Wicha M J C S C. Therapeutic implications of cellular heterogeneity and plasticity in breast cancer. Cell Stem Cell (2015) 17:260–71. doi: 10.1016/j.stem.2015.08.014 PMC4560840 26340526

[4] Zardavas D Irrthum A Swanton C Piccart M J N R C O. Clinical management of breast cancer heterogeneity. Nat Rev Clin Oncol (2015) 12:381–94. doi: 10.1038/nrclinonc.2015.73 25895611

[5] Zhao N Rosen J J S I CB. Breast cancer heterogeneity through the lens of single-cell analysis and spatial pathologies. Semin Cancer Biol(2022) 82:3–10. doi: 10.1016/j.semcancer.2021.07.010 34274486 PMC8761219

[6] Tsvetkov P Coy S Petrova B Dreishpoon M Verma A Abdusamad M. Copper induces cell death by targeting lipoylated TCA cycle proteins. Science (2022) 375:1254–61. doi: 10.1126/science.abf0529 PMC9273333 35298263

[7] Tsvetkov P Detappe A Cai K Keys H Brune Z Ying W. Mitochondrial metabolism promotes adaptation to proteotoxic stress. Nat Chem Biol (2019) 15:681–9. doi: 10.1038/s41589-019-0291-9 PMC8183600 31133756

[8] Topalian S Hodi F Brahmer J Gettinger S Smith D McDermott D. Safety, activity, and immune correlates of anti-PD-1 antibody in cancer. N Engl J Med (2012) 366:2443–54. doi: 10.1056/NEJMoa1200690 PMC3544539 22658127

[9] Vitale I Shema E Loi S Galluzzi LJNM. Intratumoral heterogeneity in cancer progression and response to immunotherapy. Nat Med (2021) 27:212–24. doi: 10.1038/s41591-021-01233-9 33574607

[10] Denkert C von Minckwitz G Darb-Esfahani S Lederer B Heppner B Weber K. Tumour-infiltrating lymphocytes and prognosis in different subtypes of breast cancer: a pooled analysis of 3771 patients treated with neoadjuvant therapy. Lancet Oncol (2018) 19:40–50. doi: 10.1016/s1470-2045(17)30904-x 29233559

[11] Loi S Michiels S Salgado R Sirtaine N Jose V Fumagalli D. Tumor infiltrating lymphocytes are prognostic in triple negative breast cancer and predictive for trastuzumab benefit in early breast cancer: results from the FinHER trial. Ann Oncol (2014) 25:1544–50. doi: 10.1093/annonc/mdu112 24608200

[12] Lehmann B Bauer J Chen X Sanders M Chakravarthy A Shyr Y. Identification of human triple-negative breast cancer subtypes and preclinical models for selection of targeted therapies. J Clin Invest (2011) 121:2750–67. doi: 10.1172/jci45014 PMC3127435 21633166

[13] Stanton S Disis M J J F I O C. Clinical significance of tumor-infiltrating lymphocytes in breast cancer. J Immunother Cancer (2016) 4:59. doi: 10.1186/s40425-016-0165-6 27777769 PMC5067916

[14] Pruneri G Vingiani A Denkert CJB. Tumor infiltrating lymphocytes in early breast cancer. Breast (2018) 37:207–14. doi: 10.1016/j.breast.2017.03.010 28363679

[15] Quail D Joyce JJNM. Microenvironmental regulation of tumor progression and metastasis. Nat Med (2013) 19:1423–37. doi: 10.1038/nm.3394 PMC3954707 24202395

[16] Binnewies M Roberts E Kersten K Chan V Fearon D Merad M. Understanding the tumor immune microenvironment (TIME) for effective therapy. Nat Med (2018) 24:541–50. doi: 10.1038/s41591-018-0014-x PMC5998822 29686425

[17] Liu J Hong M Li Y Chen D Wu Y Hu Y J F I I. Programmed cell death tunes tumor immunity. Front Immunol (2022) 13:847345. doi: 10.3389/fimmu.2022.847345 35432318 PMC9005769

[18] Niu X Chen L Li Y Hu Z He F J S I C B. Ferroptosis, necroptosis, and pyroptosis in the tumor microenvironment: Perspectives for immunotherapy of SCLC. Semin Cancer Biol (2022). doi: 10.1016/j.semcancer.2022.03.009 35288298

[19] Allensworth J Evans M Bertucci F Aldrich A Festa R Finetti P. Disulfiram (DSF) acts as a copper ionophore to induce copper-dependent oxidative stress and mediate anti-tumor efficacy in inflammatory breast cancer. Mol Oncol (2015) 9:1155–68. doi: 10.1016/j.molonc.2015.02.007 PMC4493866 25769405

[20] Chen D Cui Q Yang H Dou QJCR. Disulfiram, a clinically used anti-alcoholism drug and copper-binding agent, induces apoptotic cell death in breast cancer cultures and xenografts via inhibition of the proteasome activity. Cancer Res (2006) 66:10425–33. doi: 10.1158/0008-5472.Can-06-2126 17079463

[21] Skrott Z Mistrik M Andersen K Friis S Majera D Gursky J. Alcohol-abuse drug disulfiram targets cancer *via* p97 segregase adaptor NPL4. Nature (2017) 552:194–9. doi: 10.1038/nature25016 PMC5730499 29211715

[22] Yip N Fombon I Liu P Brown S Kannappan V Armesilla A. Disulfiram modulated ROS-MAPK and NFκB pathways and targeted breast cancer cells with cancer stem cell-like properties. Br J Cancer (2011) 104:1564–74. doi: 10.1038/bjc.2011.126 PMC3101904 21487404

[23] Sun T Yang W Toprani S Guo W He L DeLeo A. Induction of immunogenic cell death in radiation-resistant breast cancer stem cells by repurposing anti-alcoholism drug disulfiram. Cell Commun Signal (2020) 18:36. doi: 10.1186/s12964-019-0507-3 32138738 PMC7057578

[24] Liu J Lichtenberg T Hoadley K Poisson L Lazar A Cherniack A. An integrated TCGA pan-cancer clinical data resource to drive high-quality survival outcome analytics. Cell (2018) 173:400–416.e11. doi: 10.1016/j.cell.2018.02.052 29625055 PMC6066282

[25] Șenbabaoğlu Y Michailidis G Li JJSR. Critical limitations of consensus clustering in class discovery. Sci Rep (2014) 4:6207. doi: 10.1038/srep06207 25158761 PMC4145288

[26] Charoentong P Finotello F Angelova M Mayer C Efremova M Rieder D. Pan-cancer immunogenomic analyses reveal genotype-immunophenotype relationships and predictors of response to checkpoint blockade. Cell Rep (2017) 18:248–62. doi: 10.1016/j.celrep.2016.12.019 28052254

[27] Yu G Wang LG Han Y. And he q y clusterProfiler: An r package for comparing biological themes among gene clusters. Omics (2012) 16:284–7. doi: 10.1089/omi.2011.0118 PMC3339379 22455463

[28] Gendoo DM Ratanasirigulchai N Schröder MS Paré L Parker JS Prat A. Genefu: An R/Bioconductor package for computation of gene expression-based signatures in breast cancer. Bioinformatics (2016) 32:1097–9. doi: 10.1093/bioinformatics/btv693 PMC6410906 26607490

[29] Mayakonda A Lin D Assenov Y Plass C Koeffler HJGR. Maftools: Efficient and comprehensive analysis of somatic variants in cancer. Genome Res (2018) 28:1747–56. doi: 10.1101/gr.239244.118 PMC6211645 30341162

[30] Chen D Mellman IJN. Elements of cancer immunity and the cancer-immune set point. Nature (2017) 541:321–30. doi: 10.1038/nature21349 28102259

[31] Herbst RS Soria JC Kowanetz M Fine GD Hamid O Gordon MS. Predictive correlates of response to the anti-PD-L1 antibody MPDL3280A in cancer patients. Nature (2014) 515:563–7. doi: 10.1038/nature14011 PMC4836193 25428504

[32] Tumeh P Harview C Yearley J Shintaku I Taylor E Robert L. PD-1 blockade induces responses by inhibiting adaptive immune resistance. Nature (2014) 515:568–71. doi: 10.1038/nature13954 PMC4246418 25428505

[33] Rosenberg J Hoffman-Censits J Powles T van der Heijden M Balar A Necchi A. Atezolizumab in patients with locally advanced and metastatic urothelial carcinoma who have progressed following treatment with platinum-based chemotherapy: A single-arm, multicentre, phase 2 trial. Lancet (2016) 387:1909–20. doi: 10.1016/s0140-6736(16)00561-4 PMC5480242 26952546

[34] Qian C Cao X J S I I. Dendritic cells in the regulation of immunity and inflammation. Semin Immunol (2018) 35:3–11. doi: 10.1016/j.smim.2017.12.002 29242034

[35] Wculek S Cueto F Mujal A Melero I Krummel M Sancho D J N R I. Dendritic cells in cancer immunology and immunotherapy. Nat Rev Immunol (2020) 20:7–24. doi: 10.1038/s41577-019-0210-z 31467405

[36] Hessmann E Buchholz S Demir I Singh S Gress T Ellenrieder V. Microenvironmental determinants of pancreatic cancer. Physiol Rev (2020) 100:1707–51. doi: 10.1152/physrev.00042.2019 32297835

[37] Junttila M De Sauvage FJN. Influence of tumour micro-environment heterogeneity on therapeutic response. Nature (2013) 501:346–54. doi: 10.1038/nature12626 24048067

[38] Sorokin L J N R I. The impact of the extracellular matrix on inflammation. Nat Rev Immunol (2010) 10:712–23. doi: 10.1038/nri2852 20865019

[39] McCarthy A Barlow W Conant E Haas J Li C Sprague B. Breast cancer with a poor prognosis diagnosed after screening mammography with negative results. JAMA Oncol (2018) 4:998–1001. doi: 10.1001/jamaoncol.2018.0352 29801067 PMC6145719

[40] Wellenstein M Coffelt S Duits D van Miltenburg M Slagter M de Rink I. Loss of p53 triggers WNT-dependent systemic inflammation to drive breast cancer metastasis. Nature (2019) 572:538–42. doi: 10.1038/s41586-019-1450-6 PMC6707815 31367040

[41] Ghosh M Saha S Bettke J Nagar R Parrales A Iwakuma T. Mutant p53 suppresses innate immune signaling to promote tumorigenesis. Cancer Cell (2021) 39:494–508.e5. doi: 10.1016/j.ccell.2021.01.003 33545063 PMC8044023

[42] Shah S Roth A Goya R Oloumi A Ha G Zhao Y. The clonal and mutational evolution spectrum of primary triple-negative breast cancers. Nature (2012) 486:395–9. doi: 10.1038/nature10933 PMC3863681 22495314

[43] Koboldt DC Fulton RS McLellan MD Schmidt H Kalicki-Veizer J McMichael JF . Comprehensive molecular portraits of human breast tumours. Nature (2012) 490:61–70. doi: 10.1038/nature11412 23000897 PMC3465532

[44] Turley S Cremasco V Astarita J J N R I. Immunological hallmarks of stromal cells in the tumour microenvironment. Nat Rev Immunol (2015) 15:669–82. doi: 10.1038/nri3902 26471778

[45] Mittendorf EA Philips AV Meric-Bernstam F Qiao N Wu Y Harrington S. PD-L1 expression in triple-negative breast cancer. Cancer Immunol Res (2014) 2:361–70. doi: 10.1158/2326-6066.Cir-13-0127 PMC4000553 24764583

[46] Luen S Virassamy B Savas P Salgado R Loi SJB. The genomic landscape of breast cancer and its interaction with host immunity. Breast (2016) 29:241–50. doi: 10.1016/j.breast.2016.07.015 27481651

[47] Doroshow D Bhalla S Beasley M Sholl L Kerr K Gnjatic S. PD-L1 as a biomarker of response to immune-checkpoint inhibitors. Nat Rev Clin Oncolc (2021) 18:345–62. doi: 10.1038/s41571-021-00473-5 33580222

[48] Schmid P Adams S Rugo HS Schneeweiss A Barrios CH Iwata H. Atezolizumab and nab-paclitaxel in advanced triple-negative breast cancer. N Engl J Med (2018) 379:2108–21. doi: 10.1056/NEJMoa1809615 30345906

