# Supplementary figures and images for: Identification of cuproptosis-related patterns and construction of a scoring system for predicting prognosis, tumor microenvironment-infiltration characteristics, and immunotherapy efficacy in breast cancer
Source: Front Oncol. 2022 Sep 23;12:966511. doi: 10.3389/fonc.2022.966511 (PMC9544817; doi:10.3389/fonc.2022.966511)

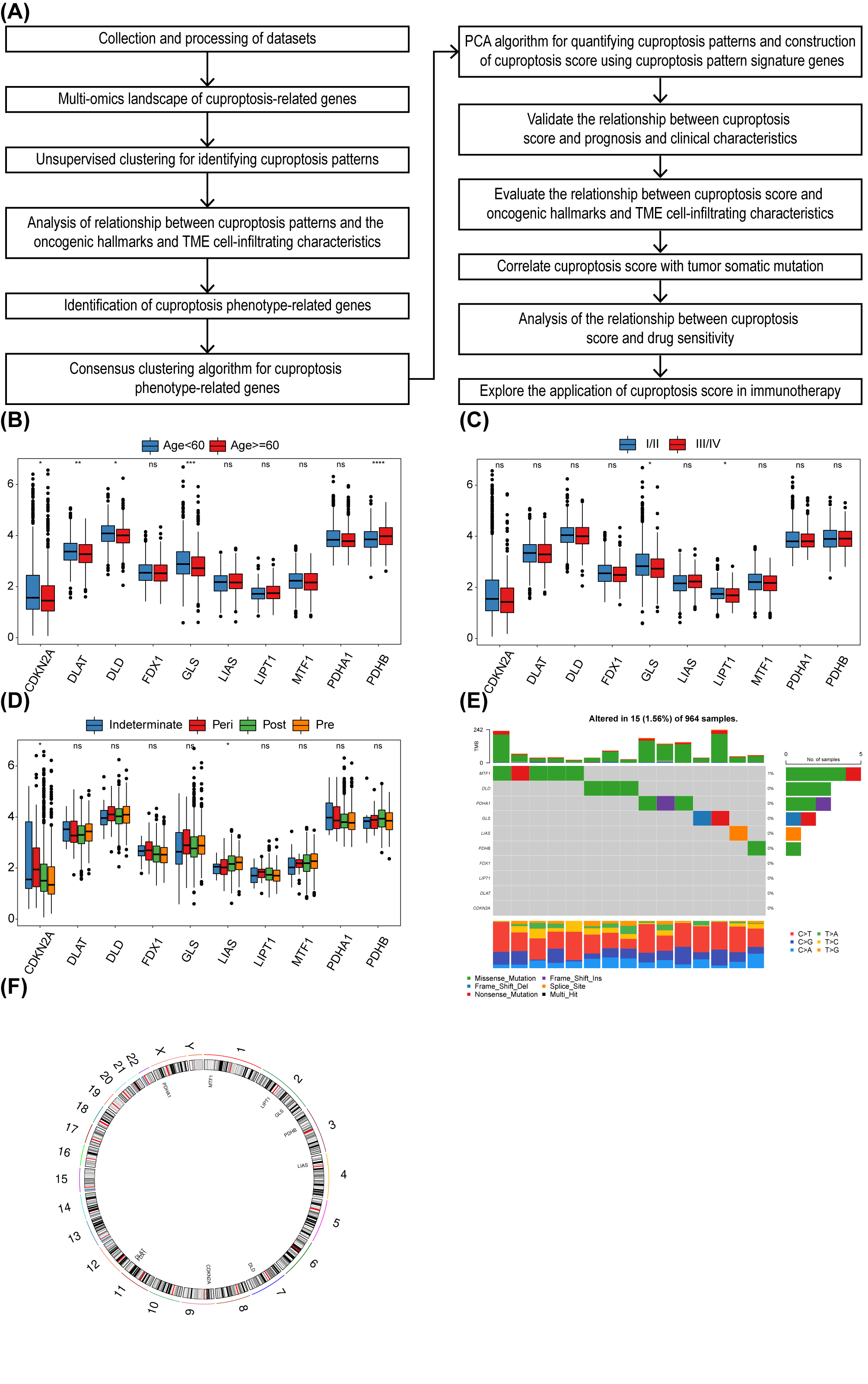

Supplement: Supplementary Figure 1 — Expression of CRGs in different clinical characteristic subgroups and genetic alterations. (A) Overview of this study. (B) Boxplot shows the expression of 10 CRGs between different age groups in the TCGA-BRCA cohort. Age< 60, blue; Age ≧60, red. (C) Boxplot shows the expression of 10 CRGs between different stage groups in the TCGA-BRCA cohort. Stage I/II, blue; Stage III/IV, red. (D) Boxplot shows the expression of 10 CRGs between different menopause groups in the TCGA-BRCA cohort. Indeterminate, blue; Peri, red; Post, green; Pre, orange. (E) The mutation frequency of 10 CRGs in patients from TCGA-BRCA cohort. (F) The location of 10 CRGs on 23 chromosomes. (ns, P ≥ 0.05, * P< 0.05, ** P< 0.01, *** P< 0.001, and **** P< 0.0001) [file Image_1.tif]

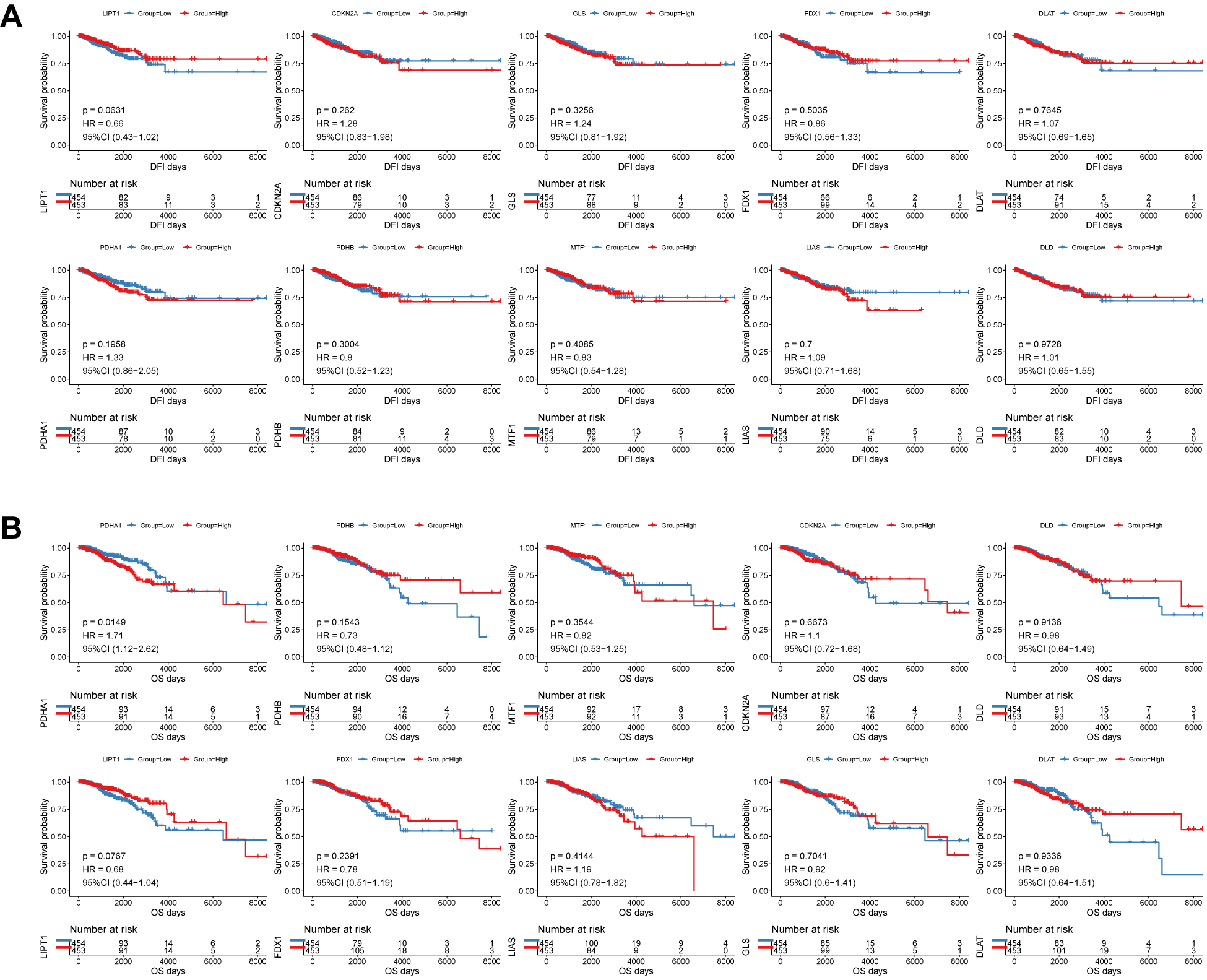

Supplement: Supplementary Figure 2 — Survival analyses for patients with low and high CRGs expression. [file Image_2.tif]

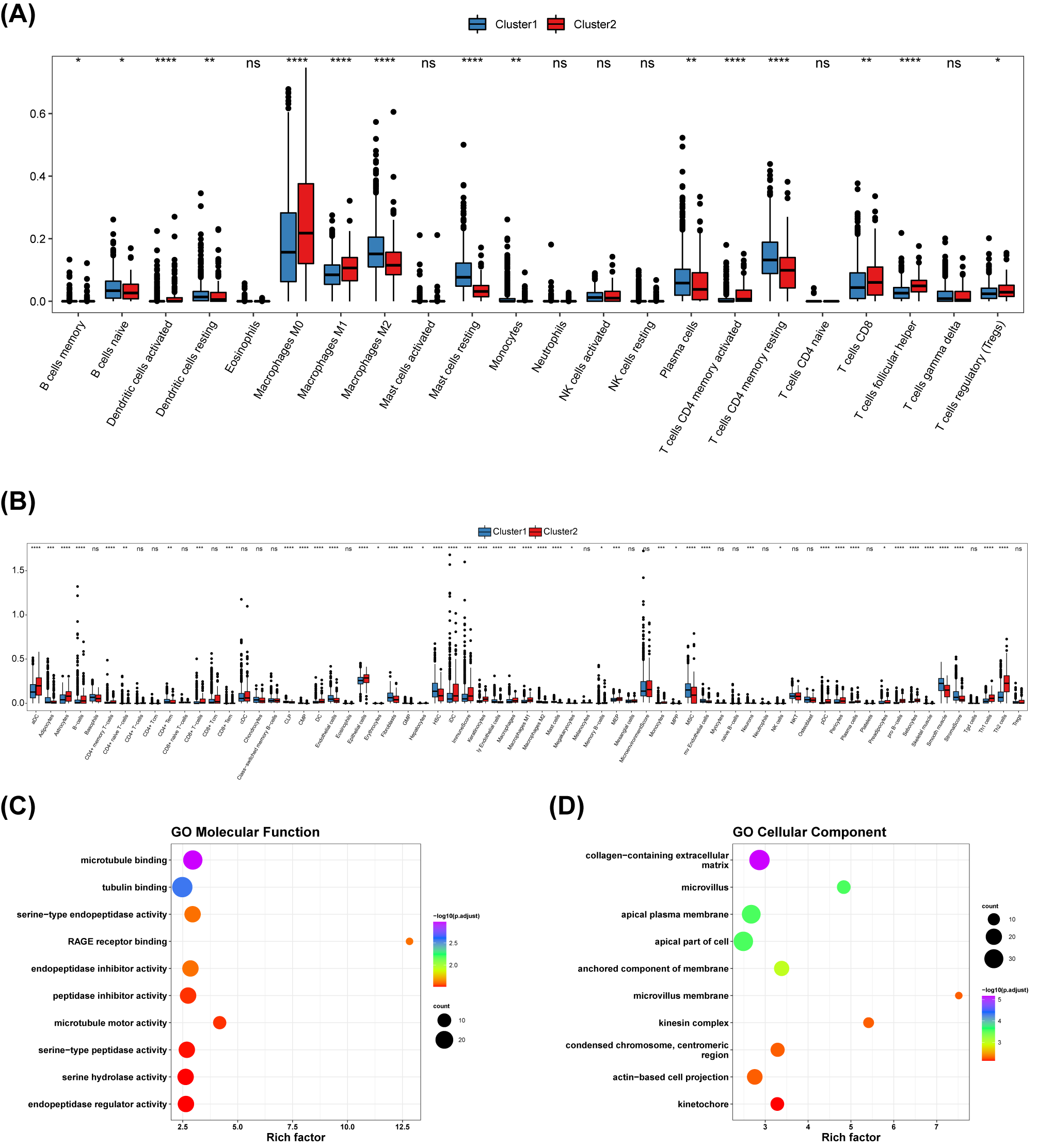

Supplement: Supplementary Figure 3 — TME characteristics and biological features of cuproptosis patterns. (A) The abundance of each TME infiltrating cell in cuproptosis patterns. (B) The proportion of TME infiltration cell in cuproptosis patterns. (C, D) Molecular function and cellular component annotation for cuproptosis phenotype-related DEGs using GO enrichment analysis. (ns, P ≥ 0.05, * P< 0.05, ** P< 0.01, *** P< 0.001, and **** P< 0.0001) [file Image_3.tif]

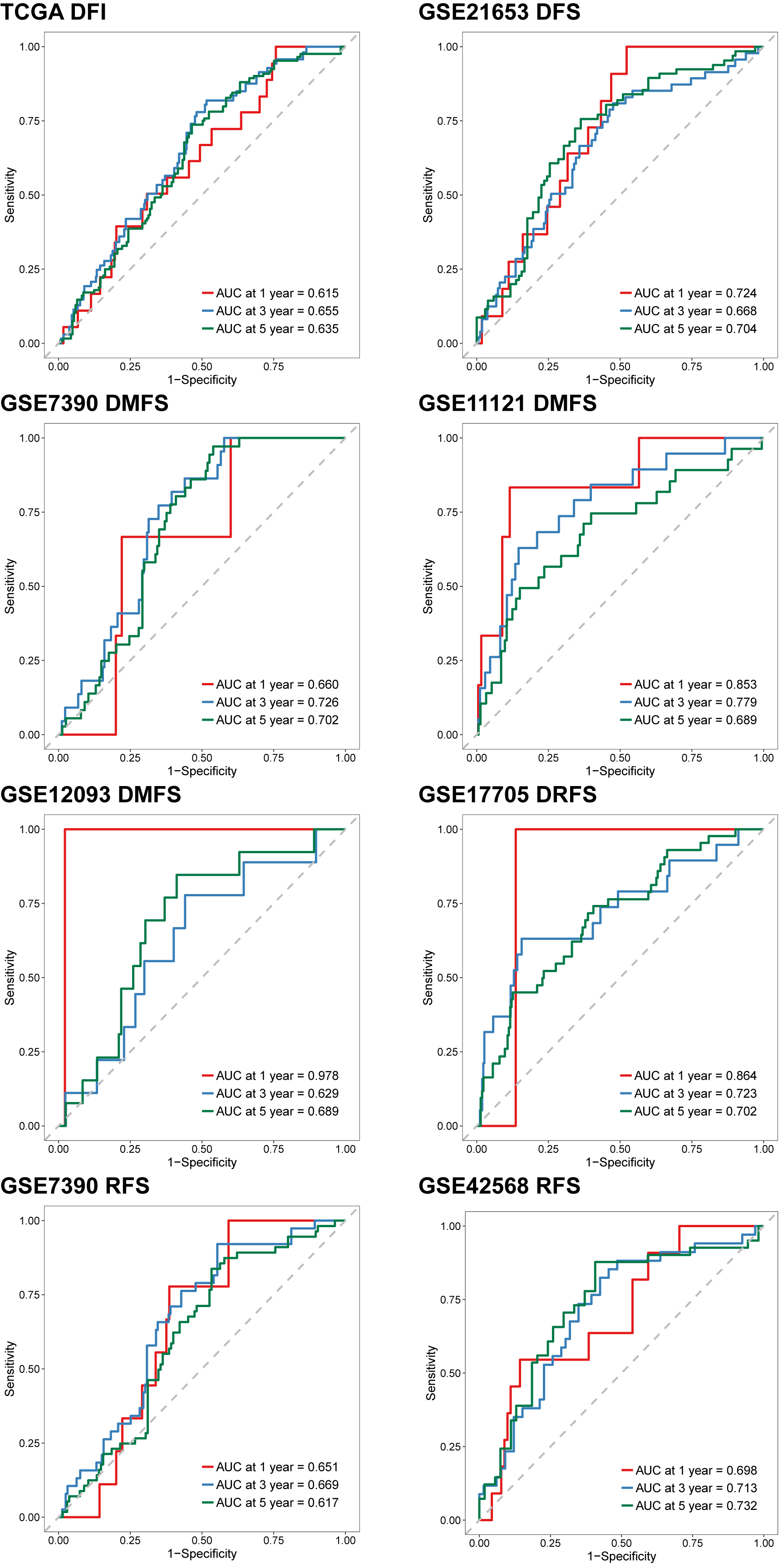

Supplement: Supplementary Figure 4 — The predictive value of cuproptosis score in training and validation cohorts. [file Image_4.tif]

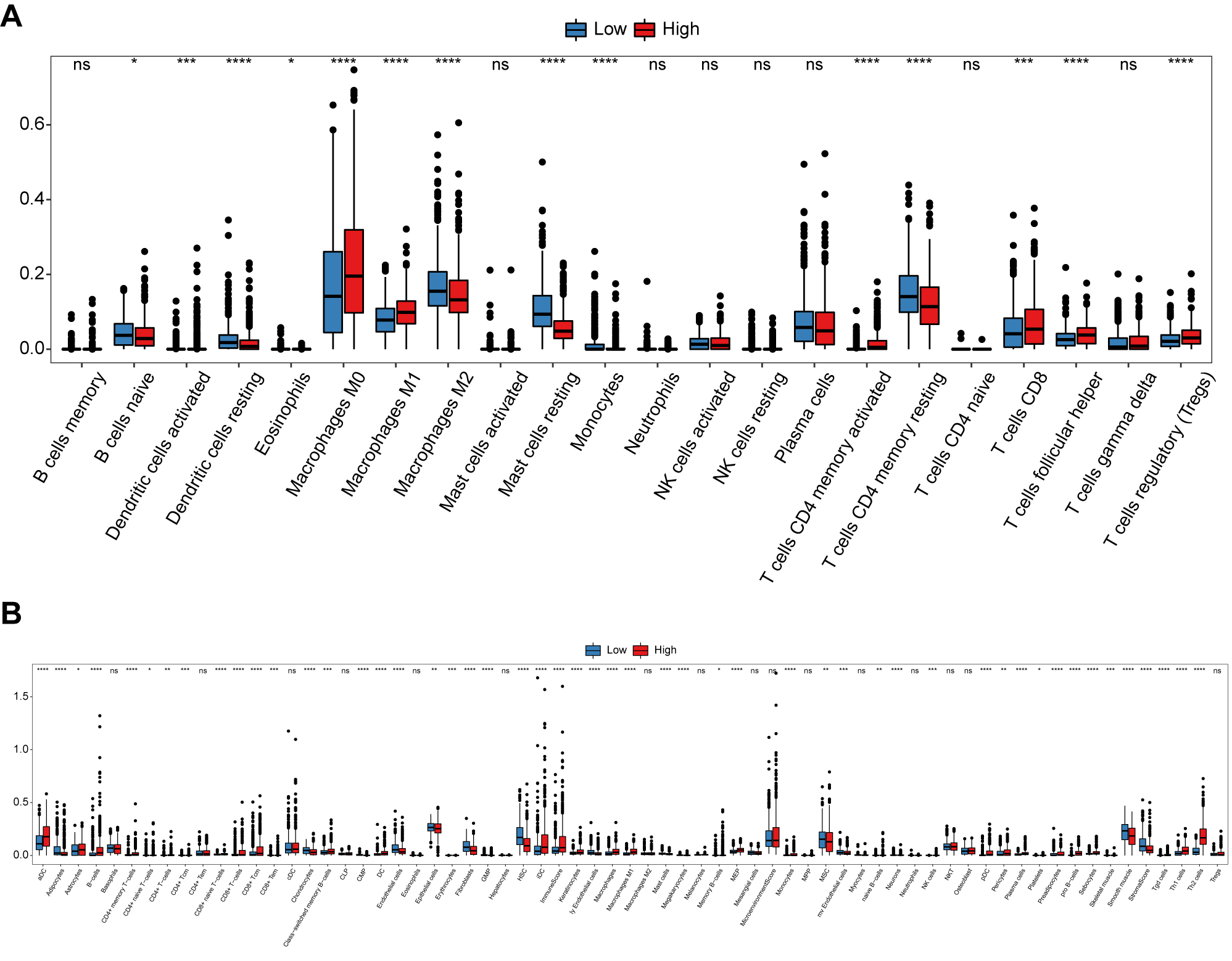

Supplement: Supplementary Figure 5 — TME characteristics of high and low cuproptosis score groups. (A) The abundance of each TME infiltrating cell in high and low cuproptosis score groups. (B) The proportion of TME infiltration cell in high and low cuproptosis score groups. (ns, P ≥ 0.05, * P< 0.05, ** P< 0.01, *** P< 0.001, and **** P< 0.0001) [file Image_5.tif]

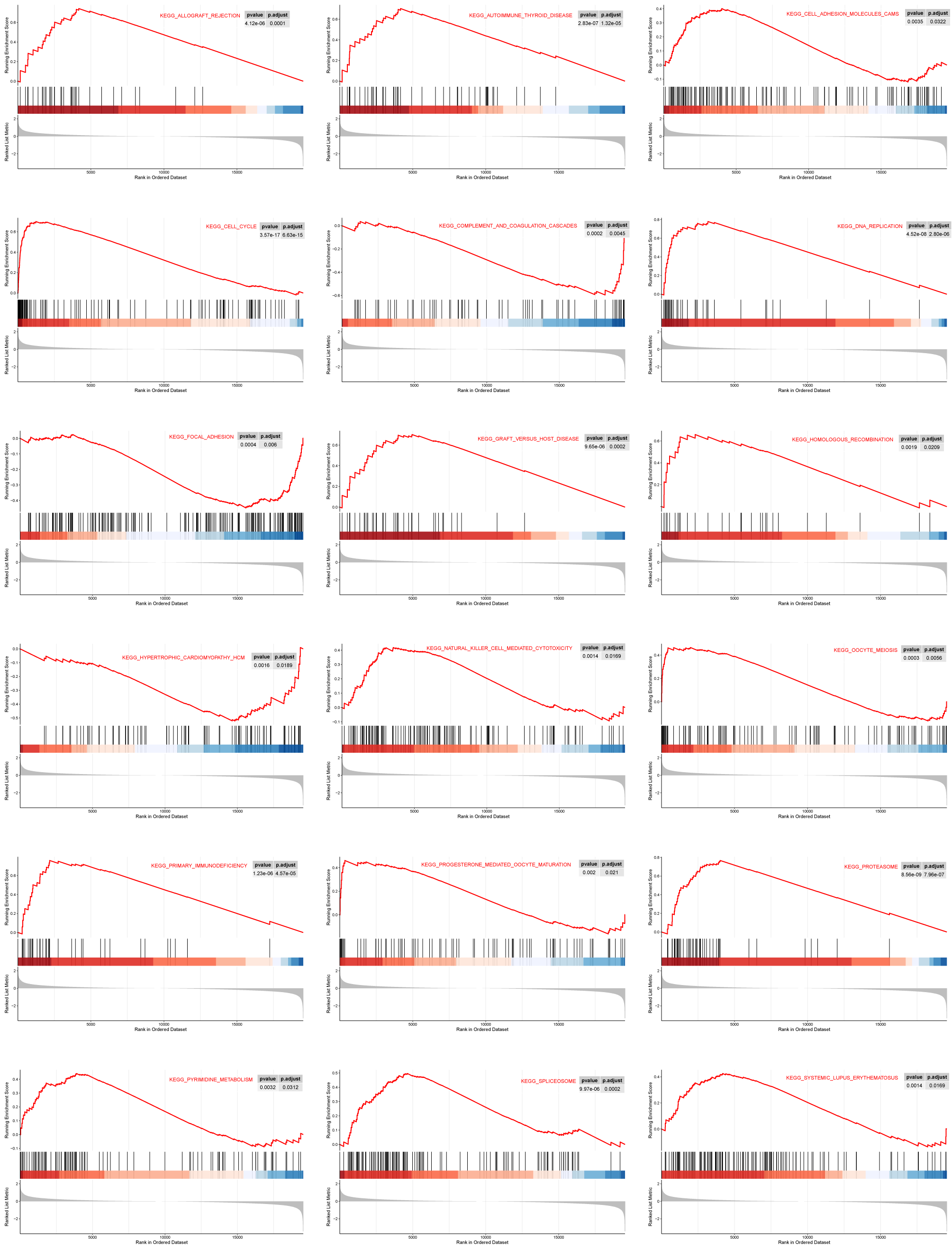

Supplement: Supplementary Figure 6 — The GSEA enrichment analysis in terms of the high and low cuproptosis score groups. [file Image_6.tif]
